# A MST1–FOXO1 cascade establishes endothelial tip cell polarity and facilitates sprouting angiogenesis

**DOI:** 10.1038/s41467-019-08773-2

**Published:** 2019-02-19

**Authors:** Yoo Hyung Kim, Jeongwoon Choi, Myung Jin Yang, Seon Pyo Hong, Choong-kun Lee, Yoshiaki Kubota, Dae-Sik Lim, Gou Young Koh

**Affiliations:** 1 Graduate School of Medical Science and Engineering, Korea Advanced Institute of Science and Technology (KAIST), Daejeon, 34141 Korea; 2 Biomedical Science and Engineering Interdisciplinary Program, KAIST, Daejeon, 34141 Korea; 3 Center for Vascular Research, Institute for Basic Science (IBS), Daejeon, 34141 Korea; 4 Department of Vascular Biology, The Sakaguchi Laboratory, Keio University School of Medicine, Tokyo, 160-8582 Japan; 5 National Creative Research Initiatives Center for Cell Division and Differentiation, Department of Biological Science, KAIST, Daejeon, 34141 Korea

## Abstract

Hypoxia is a main driver of sprouting angiogenesis, but how tip endothelial cells are directed to hypoxic regions remains poorly understood. Here, we show that an endothelial MST1–FOXO1 cascade is essential for directional migration of tip cells towards hypoxic regions. In mice, endothelial‐specific deletion of either MST1 or FOXO1 leads to the loss of tip cell polarity and subsequent impairment of sprouting angiogenesis. Mechanistically, MST1 is activated by reactive oxygen species (ROS) produced in mitochondria in response to hypoxia, and activated MST1 promotes the nuclear import of FOXO1, thus augmenting its transcriptional regulation of polarity and migration‐associated genes. Furthermore, endothelial MST1‐FOXO1 cascade is required for revascularization and neovascularization in the oxygen-induced retinopathy model. Together, the results of our study delineate a crucial coupling between extracellular hypoxia and an intracellular ROS‐MST1‐FOXO1 cascade in establishing endothelial tip cell polarity during sprouting angiogenesis.

## Abstract (web summary)

Angiogenesis is driven by the directed migration of tip endothelial cells towards hypoxic tissues. Here, Kim et al. show that the generation of reactive oxygen species in endothelial cells upon hypoxia activates MST1, which subsequently promotes the nuclear translocation of FOXO1, and thus activates a pro-migratory transcriptional programme in endothelial tip cells.

## Introduction

The vascular system expands its network from pre-existing vessels by sprouting angiogenesis for supplying oxygen and nutrients to avascular and hypoxic tissues. In response to numerous angiogenic cues from oxygen- and nutrient-deprived tissues, endothelial cells (ECs), the main components of the vascular lumen, adopt a series of morphogenic behaviors, such as tip ECs and stalk ECs, for coordinating sprouting angiogenesis^1–3^. Tip ECs are championed cells and highly migratory, leading the sprouts in the direction of a guidance cue, while stalk ECs are proliferative, supplying building blocks for sprout elongation^1,2,4^. Haemodynamic forces drive lumen expansion into newly formed sprouts to deliver oxygen- and nutrient-rich blood flow^5,6^. These overall processes are finely regulated by various extrinsic cues and corresponding intrinsic signaling in the ECs. Lately, significant advances have been made in the understanding of intrinsic transcriptional and metabolic changes in tip ECs^7–11;^ however, how they are directed—the EC polarization at the vascular front—into the avascular, hypoxic area is poorly understood.

Mammalian sterile 20-like kinases 1 and 2 (MST1/2) have been identified as mediators of oxidative stress^12,13^ and lately characterized as the major component of the Hippo pathway^14,15^. The mammalian core Hippo pathway components encompass MST1/2, large tumor suppressor homolog 1 and 2 (LATS1/2), and Yes-associated protein (YAP) or its paralog transcriptional coactivator with PDZ-binding motif (TAZ). YAP/TAZ are transcription coactivators that mainly interact with the TEAD/TEF family of transcription factors and play crucial roles in regulating cellular proliferation, differentiation and migration, tissue growth, and organ morphogenesis^14,15^. We and others lately have found that YAP/TAZ play critical roles in the morphogenesis of tip ECs and proliferation of stalk ECs by regulating cytoskeletal rearrangement and metabolic activity during sprouting angiogenesis^10,16–18^. LATS1/2 are direct upstream regulators of YAP/TAZ, limiting their activities through phosphorylation-dependent cytoplasmic retention and destabilization^14,15^. Indeed, endothelial deletion of LATS1/2 enhances activities of YAP/TAZ, leading to a dense and hyperplastic network, uncoordinated outgrowth, numerous filopodia bursts in tip ECs, and increased proliferating ECs in growing retinal vessels^10^. Overall, this LATS1/2-YAP/TAZ cascade responds to vascular endothelial growth factor-A (i.e., VEGF) and regulates angiogenesis^10,16^. MST1/2 are serine/threonine kinases that are expressed ubiquitously in most tissues and cell types^12–14,19^. MST1/2 phosphorylate and activate LATS1/2, and thereby inactivate YAP/TAZ in the canonical Hippo pathway. However, these kinase–substrate relationships are highly cell type- and context-dependent^19–25^. Specifically, MST1 is activated by cellular stress such as ultraviolet radiation, serum starvation, hydrogen peroxide, and reactive oxygen species (ROS)^26^, followed by phosphorylation of its cellular substrates including Forkhead box (FOXO) proteins^13,19,21,22^. In fact, MST1 mediates oxidative stress-induced neuronal cell death through phosphorylation of FOXO1 at serine 212, which leads to disruption of the association between FOXO1 and 14-3-3 proteins, subsequently enhancing nuclear import of FOXO1^19^. Of importance in ECs, FOXO1 is a crucial gatekeeper for EC quiescence mediated through reducing glycolysis, mitochondrial respiration, and proliferation by suppressing MYC during sprouting angiogenesis^11^.

Here, we unveil that MST1 acts as an upstream regulator of FOXO1 rather than of LATS1/2 and plays key roles in sprouting angiogenesis by establishing endothelial polarity at tip ECs. Moreover, hypoxia rather than VEGF monopolizes the MST1–FOXO1 cascade in this context. Our results demonstrate a crucial coupling between extracellular hypoxia and an intracellular MST1–FOXO1 cascade, which facilitates sprouting angiogenesis.

## Results

### MST1 is involved in establishing endothelial polarization

**Fig. 1:**
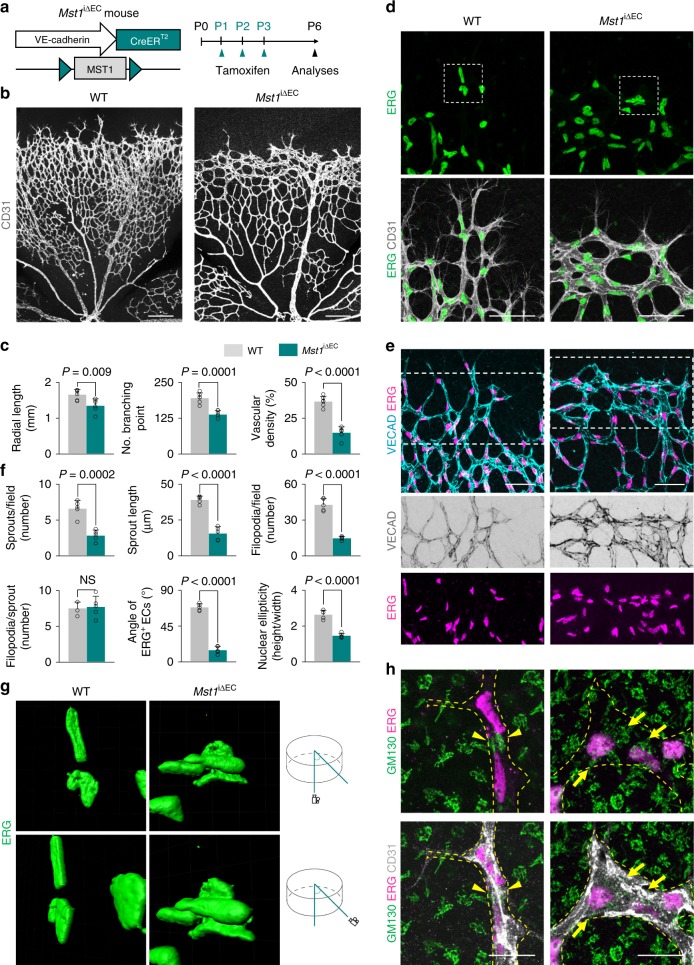
MST1 plays a critical role in establishing endothelial polarization. **a** Diagram depicting the experimental schedule for endothelial cell (EC)-specific deletion of MST1 in retinal vessels from P1 and their analyses at P6 using *Mst1*^i∆EC^ mice. **b**, **c** Images of CD31^+^ retinal vessels and comparisons of indicated parameters in WT (*n* = 6) and *Mst1*^i∆EC^ (*n* = 6) mice. Scale bars, 200 μm. **d** Magnified images of CD31^+^ vessels and ERG^+^ nuclei of ECs in WT and *Mst1*^i∆EC^ mice. The insets (white dashed-line boxes) are 3D reconstructed and magnified in **g**. Scale bars, 50 μm. **e** Images showing abnormally aligned VE-cadherin (VECAD) and ERG^+^ nuclei of ECs in the vascular front of *Mst1*^i∆EC^ mice compared with those of WT mice. Middle and bottom panels show VECAD and ERG signals of insets (white dashed-line boxes) in top panels. Scale bars, 100 μm. **f** Comparisons of indicated parameters in WT (*n* = 5) and *Mst1*^i∆EC^ (*n* = 5) mice. **g** 3D reconstructed images of ERG^+^ nuclei of ECs from the front and a 45° angle showing that the ERG^+^ nuclei of ECs overlap each other in *Mst1*^i∆EC^ mice. **h** Images of CD31^+^ vessels, ERG^+^ nuclei of ECs, and GM130^+^ Golgi apparatus at tip ECs of WT and *Mst1*^i∆EC^ mice. The yellow dashed line outlines CD31^+^ vessels. Note that GM130^+^ Golgi apparatus are polarized towards the anterior or posterior of the nuclei in tip ECs of WT mice (yellow arrowheads), while such polarization is lost in tip ECs of *Mst1*^i∆EC^ mice (yellow arrows). Scale bars, 20 μm. Data represent mean (bar) ± s.d. (error bars). *P* values, versus WT by two-tailed unpaired *t*-test. NS not significant. Source data are provided as a Source Data file

Considering that MST1/2 are upstream regulators of LATS1/2 in the Hippo pathway, we hypothesized that the roles of MST1/2 could be similar to those of LATS1/2 but opposite to those of YAP/TAZ in sprouting angiogenesis, based on our previous report^10^. To investigate the role of MST1 in sprouting angiogenesis, we generated *Mst1*^i∆EC^ mice by crossing *Mst1*^flox/flox^ mice^27^ with *VE-cadherin* Cre-ER^T2^ mice^28^ (Fig. 1a), confirmed EC-specific deletion of MST1 in these animals (Supplementary Fig. 1a–f), and examined retinal angiogenesis during the postnatal period. Cre-ER^T2^-positive but flox/flox-negative mice among the littermates for each experiment were defined as wild-type (WT) mice. EC-specific deletion of MST1 in *Mst1*^i∆EC^ mice from P1 led to impaired retinal angiogenesis at P6. Compared with WT mice, *Mst1*^i∆EC^ mice exhibited respectively 29 and 60% reduced vascular branching and density, and 72% reduced 5-ethynyl-2′-deoxyuridine (EdU) incorporation (proliferation), but no difference in cleaved caspase3 (apoptosis) in the ECs at the vascular front (Fig. 1b, c and Supplementary Fig. 2a–c). Moreover, tip ECs at the vascular front of *Mst1*^i∆EC^ mice exhibited reduced both sprout number and length, but no difference in filopodia formation per sprout compared with WT mice (Fig. 1d, f). Of special interest, *Mst1*^i∆EC^ mice showed abnormally aligned VE-cadherin (marking endothelial junctions) and ETS-related gene (ERG, marking endothelial nuclei) in the vascular front of the growing retinal vessels (Fig. 1e).

These findings led us to ask whether MST1 regulates endothelial polarization during sprout elongation. To address this question, we further analyzed shapes and locations of EC nuclei in the vascular front by visualizing ERG; these nuclei are elliptical in actively migrating cells and spherical in static cells^29^. Consistent with previous reports^7^, the nuclei of tip ECs were largely elliptical in WT mice. In contrast, they were spherical and not directed into the avascular area although filopodia properly extended in *Mst1*^i∆EC^ mice (Fig. 1d, g). Moreover, ERG^+^ nuclei of tip ECs overlapped each other in *Mst1*^i∆EC^ mice but positioned in a planar fashion in WT mice (Fig. 1g).

We additionally assessed the orientation of Golgi apparatus in tip ECs to analyze EC polarity according to previous reports^29,30^. Of note, the Golgi apparatus were polarized towards the anterior or posterior of the nuclei in tip ECs of WT mice, whereas such polarization was lost in tip ECs of *Mst1*^i∆EC^ mice (Fig. 1h and Supplementary Fig. 2d). These data indicate that MST1 plays a critical role in endothelial polarization during sprouting angiogenesis.

### MST1 is crucial for perpendicular vascular branching

**Fig. 2:**
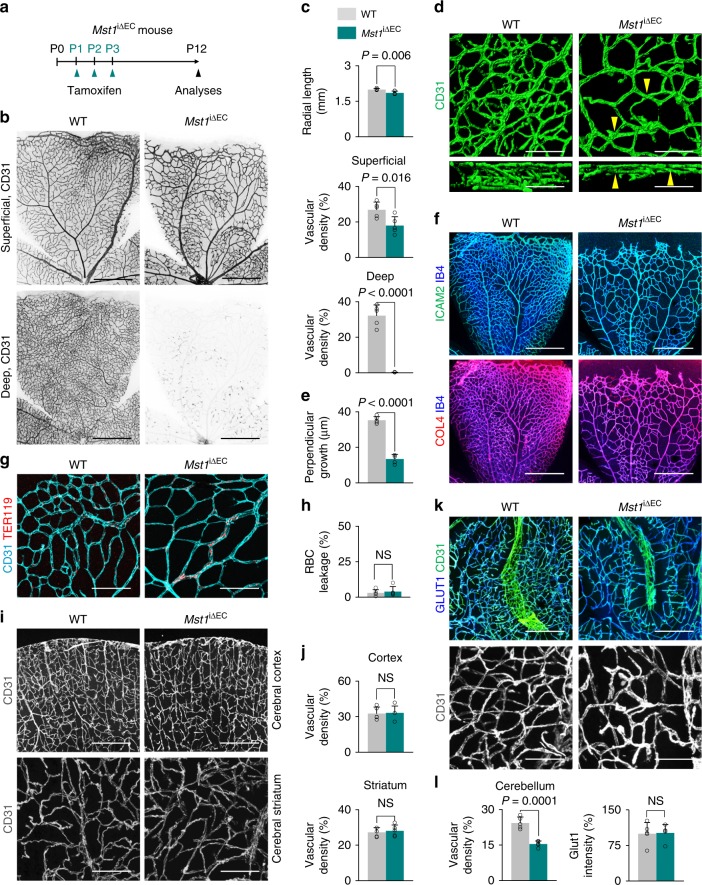
MST1 is crucial for perpendicular vascular branching. **a** Diagram depicting the experimental schedule for EC-specific deletion of MST1 in retinal vessels from P1 and their analyses at P12 in *Mst1*^i∆EC^ mice. **b**, **c** Images of CD31^+^ vessels in superficial and deep layers of retina and comparisons of indicated parameters in WT (*n* = 5) and *Mst1*^i∆EC^ (*n* = 5) mice. Scale bars, 500 μm. **d**, **e** 3D reconstructed images of CD31^+^ vascular plexus and comparisons of the length of perpendicular growth in WT (*n* = 5) and *Mst1*^i∆EC^ (*n* = 5) mice. Note that aberrant vessel crossings (yellow arrowheads) are shown. Scale bars, 50 μm. **f** Images showing IB4^+^ vessels, ICAM2^+^ lumen formation and COL4^+^ collagen deposition in WT and *Mst1*^i∆EC^ mice at P12. Scale bars, 500 μm. **g**, **h** Images of TER119^+^ RBC and CD31^+^ retinal vessels, and comparison of RBC leakage in WT (*n* = 5) and *Mst1*^i∆EC^ (*n* = 5) mice. Scale bars, 100 μm. **i**, **j** Images of CD31^+^ vessels and comparison of vascular density at cerebral cortex and striatum in WT (*n* = 5) and *Mst1*^i∆EC^ (*n* = 5) mice. Scale bars, 200 μm. **k**, **l** Images of CD31^+^ vessels and comparisons of indicated parameters at cerebellum in WT (*n* = 5) and *Mst1*^i∆EC^ (*n* = 5) mice. Scale bars, 200 μm. Data represent mean (bar) ± s.d. (error bars). *P* values, versus WT by two-tailed unpaired *t*-test. NS not significant. Source data are provided as a Source Data file

Because retinal angiogenesis analyzed at P6 represents sprouting angiogenesis in two-dimensional space^31^, we determined the role of MST1 in angiogenesis in three-dimensional space by analyzing retinal deep vascular plexus formation and brain angiogenesis at P12^32^ (Fig. 2a). Of importance, perpendicular vascular sprouting was not extended into the retinal deep layer in *Mst1*^i∆EC^ mice; instead, sprouts passed each other, displaying aberrant vessel crossings (Fig. 2b–e). As a result, vascular network formation in the retinal deep layer was severely impaired in *Mst1*^i∆EC^ mice at P12. Nevertheless, no apparent impairments in lumen formation (outlined by ICAM2 distribution), collagen type IV deposition (basement membrane), or barrier integrity [shown by TER119^+^ red blood cells (RBCs) inside vascular lumen] were shown in *Mst1*^i∆EC^ mice (Fig. 2f–h). Similarly, brain angiogenesis in cerebellum but not in cerebrum was also impaired with accompanying reduced vascular density without affecting barrier integrity (no change in GLUT1 intensity) compared with WT mice at P12 (Fig. 2i–l). These results indicate that endothelial MST1 is critical for perpendicular vascular branching.

### MST1 does not rely on the Hippo pathway in angiogenesis

Because MST2 is closest to MST1 among the five members of the MST kinase family^33^, we determined whether *Mst2* null mice^34^ exhibited similar phenotypes in retinal angiogenesis during the postnatal period (Supplementary Fig. 3a). Although no apparent differences were detected in retinal angiogenesis between WT and *Mst2* null mice, the defective phenotypes of double *Mst1*^i∆EC^-*Mst2* null mice were similar to those of *Mst1*^i∆EC^ mice (Supplementary Fig. 3b, c), indicating that endothelial MST1 but not MST2 is critical in sprouting angiogenesis.

To see whether LATS1/2 are downstream regulators of MST1 in sprouting angiogenesis, we characterized the vascular phenotypes in growing retinal vessels of *Lats1/2*^i∆EC^ mice^35–37^ and compared them with those of *Mst1*^i∆EC^ mice (Supplementary Fig. 4a). However, in contrast to those of *Mst1*^i∆EC^ mice, *Lats1/2*^i∆EC^ mice exhibited 49 and 40% increased vascular density and branching, respectively. ERG^+^ nuclei of tip ECs in *Lats1/2*^i∆EC^ mice were elliptical and properly aligned in the direction of filopodia extension, which was identical to that of WT mice (Supplementary Fig. 4b, d). Furthermore, TAZ was 12-fold enriched in the retinal vessels of *Lats1/2*^i∆EC^ mice but unchanged in those of *Mst1*^i∆EC^ mice compared with WT mice (Supplementary Fig. 4c, d). Thus, vascular phenotypes of *Mst1*^i∆EC^ mice were completely different from those of *Lats1/2*^i∆EC^ mice, meaning that MST1 does not act as an upstream regulator of LATS1/2 by taking a canonical Hippo pathway for directional sprouting angiogenesis. These findings led us to seek an alternative downstream pathway of MST1 for sprouting angiogenesis.

### MST1 regulates FOXO1 localization at tip ECs

**Fig. 3:**
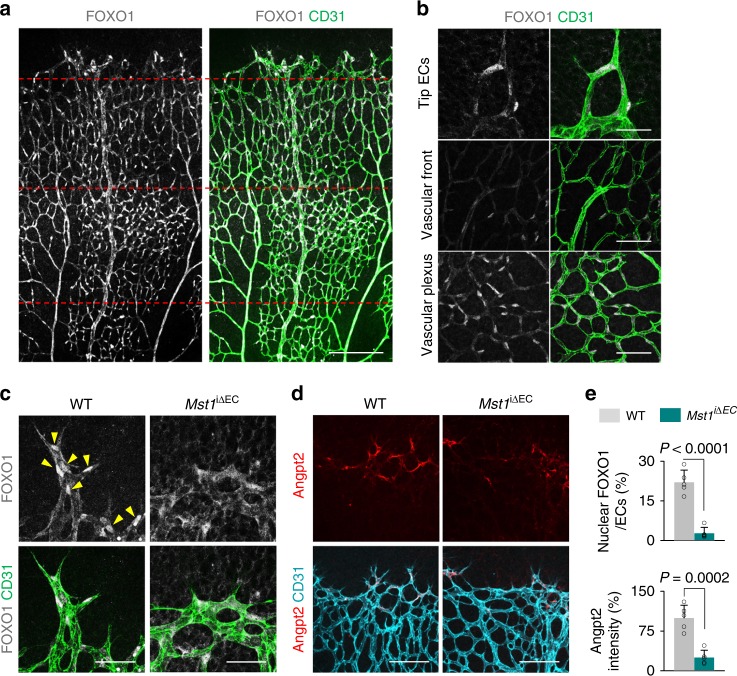
MST1 regulates nuclear localization of FOXO1 at tip ECs. **a** Images of CD31^+^ retinal vessels and distribution of FOXO1 of whole retina in WT mouse at P6. The red dashed lines separate into tip ECs, vascular front and vascular plexus from top to bottom. Scale bars, 200 μm. **b** Magnified images of CD31^+^ vessels and subcellular localization of FOXO1 at indicated portions. Scale bars, 50 μm. **c** Magnified images of the nuclear localization of FOXO1 (yellow arrowheads) at tip ECs in WT and *Mst1*^i∆EC^ mice. Scale bars, 50 μm. **d** Images of angiopoietin-2 (Angpt2) expression and CD31^+^ vessels at vascular front in WT and *Mst1*^i∆EC^ mice. Scale bars, 100 μm. **e** Comparisons of indicated parameters in WT (*n* = 5) and *Mst1*^i∆EC^ (*n* = 5) mice. Data represent mean (bar) ± s.d. (error bars). *P* values, versus WT by two-tailed unpaired *t*-test. NS not significant. Source data are provided as a Source Data file

In seeking the alternative downstream effector of MST1 beyond the canonical Hippo pathway, we predicted that FOXO1, a critical transcriptional and metabolic regulator for ECs during sprouting angiogenesis^11^, can be a downstream cellular substrate of MST1, based on the findings at other cell types in previous reports^19,21,22^. Of note, FOXO1 was highly and specifically expressed in most ECs but showed differential subcellular localizations in different regions of growing retinal blood vessels (Fig. 3a, b). Consistent with a previous report^11^, a diffuse nucleocytoplasmic localization of FOXO1 at the vascular front but relatively intense nuclear localization at the vascular plexus was shown. Beyond those findings, we identified relatively strong nuclear localization of FOXO1 at tip ECs of WT mice (Fig. 3b). However, of special note, *Mst1*^i∆EC^ mice exhibited a nucleocytoplasmic localization of FOXO1 at tip ECs. Moreover, angiopoietin-2 (Angpt2), which is expressed at tip ECs under control of FOXO1, was markedly reduced in *Mst1*^i∆EC^ mice (Fig. 3c–e). These data imply that MST1 may promote nuclear import of FOXO1 in tip ECs during sprouting angiogenesis.

### FOXO1 is required for establishing endothelial polarization

**Fig. 4:**
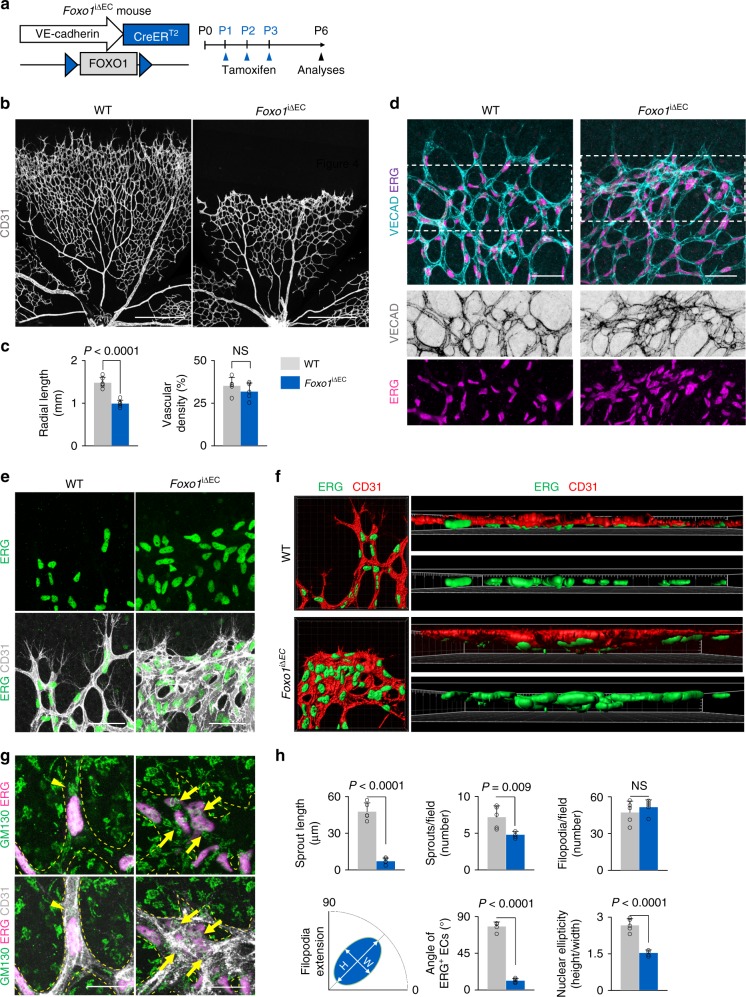
FOXO1 is required for establishing endothelial polarization. **a** Diagram depicting the experiment schedule for EC-specific deletion of FOXO1 in retinal vessels from P1 and their analyses at P6. **b**, **c** Images of CD31^+^ vessels and comparisons of indicated parameters in WT (*n* = 5) and *Foxo1*^i∆EC^ (*n* = 5) mice. Scale bar, 500 μm. **d** Images showing VECAD and ERG^+^ nuclei of ECs at the vascular front of WT and *Foxo1*^i∆EC^ mice. Middle and bottom panels show VE-cadherin (VECAD) and ERG signals of insets (dashed-line boxes) in top panels. Scale bars, 100 μm. **e** Magnified images of CD31^+^ vessels and ERG^+^ nuclei of ECs. Scale bars, 50 μm. **f** 3D reconstructed images of CD31^+^ vessels and ERG^+^ nuclei of ECs in WT and *Foxo1*^i∆EC^ mice. **g** Images of CD31^+^ vessels, ERG^+^ nuclei of ECs and GM130^+^ Golgi apparatus at tip ECs in WT and *Foxo1*^i∆EC^ mice. The yellow dashed line outlines CD31^+^ vessels. Note that GM130^+^ Golgi apparatus are polarized towards the anterior or posterior of the nuclei in tip ECs of WT mice (yellow arrowheads), while such polarization is lost in tip ECs of *Foxo1*^i∆EC^ mice (yellow arrows). Scale bars, 20 μm. **h** Comparisons of indicated parameters in WT (*n* = 5) and *Foxo1*^∆EC^ (*n* = 5) mice. Data represent mean (bar) ± s.d. (error bars). *p* values, versus WT by two-tailed unpaired *t*-test. NS not significant. Source data are provided as a Source Data file

To address the role of nuclear FOXO1 in tip ECs during sprouting elongation, we generated *Foxo1*^i∆EC^ mice by crossing *Foxo1*^flox/flox^ mice^38^ with *VE-cadherin* Cre-ER^T2^ mice (Fig. 4a and Supplementary Fig. 5a, b). When FOXO1 was specifically deleted in ECs of *Foxo1*^i∆EC^ mice from P1, the mice at P6 showed retarded radial vessel growth (Fig. 4b, c). *Foxo1*^i∆EC^ mice exhibited three major defective subtypes of vascular sprouts depending on the regionally variant subcellular localization of FOXO1 in growing retinal blood vessels: flat type, hill type at the peri-arterial ECs, and glomeruloid type at the peri-venous ECs (Supplementary Fig. 5c). However, regardless of these subtypes, abnormally aligned VE-cadherin and ERG^+^ nuclei were detected in all tip ECs of *Foxo1*^i∆EC^ mice (Fig. 4d). In fact, tip ECs were stacked with vascular front ECs and the ERG^+^ nuclei of tip ECs were spherical and randomly positioned, but the filopodia extension of tip ECs was directed to the avascular region in *Foxo1*^i∆EC^ mice (Fig. 4e, h). Of interest, the ERG^+^ nuclei of tip ECs strikingly overlapped each other in *Foxo1*^i∆EC^ mice, but arranged in a planar distribution in WT mice (Fig. 4f). Moreover, the polarization of Golgi apparatus was lost in tip ECs of *Foxo1*^i∆EC^ mice (Fig. 4g and Supplementary Fig. 5d). Thus, the vascular phenotypes of *Foxo1*^i∆EC^ mice are similar to those of *Mst1*^i∆EC^ mice, implying that FOXO1 could be a downstream substrate of MST1 for endothelial polarization during sprouting angiogenesis.

### Hypoxia activates MST1–FOXO1 cascade in primary cultured ECs

**Fig. 5:**
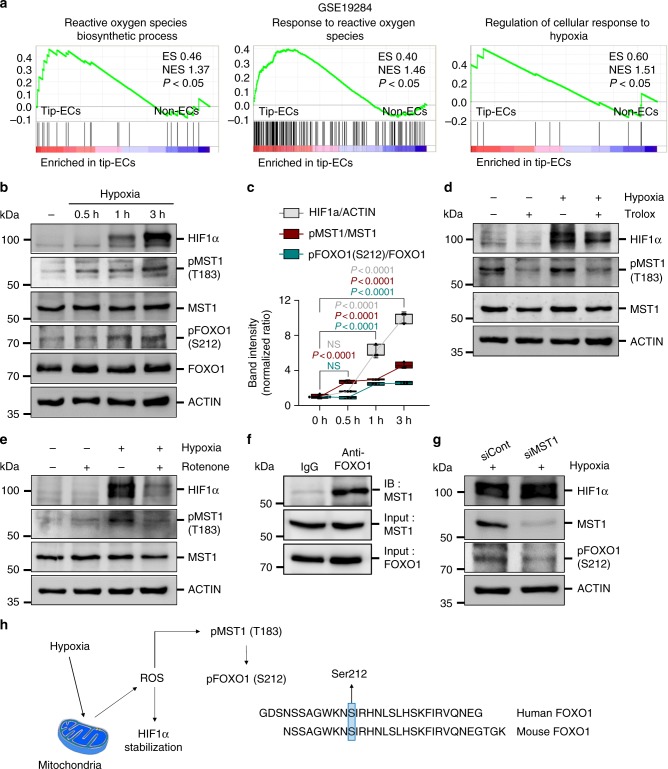
Hypoxia activates the MST1–FOXO1 cascade in primary cultured ECs. **a** GSEA analyses of the microarray data (GSE19284) obtained from isolated tip ECs and non-ECs by laser capture microdissection. **b**, **c** Immunoblot analyses and temporal changes of indicated proteins in HUVECs exposed to hypoxia (1% O_2_) for indicated times (*n* = 3, each group). Center line, median; Box limits, upper and lower quartiles; Whiskers, s.d. *p* values versus 0 h by one-way ANOVA with Tukey’s post hoc test. NS not significant. **d** Immunoblot analyses of indicated proteins in HUVECs under normoxia (−) and hypoxia (+) in absence (−) or presence (+) of Trolox treatment. **e** Immunoblot analyses of indicated proteins in HUVECs under normoxia (−) and hypoxia (+) in absence (−) or presence (+) of Rotenone treatment. **f** Immunoprecipitation analysis in HUVECs with control anti-IgG and anti-FOXO1 antibody followed by immunoblotting with anti-MST1 antibody. **g** Immunoblot analyses of indicated proteins in siCont-ECs and siMST1-ECs under hypoxia. **h** Schematic picture depicting a hypoxia-intracellular ROS–MST1–FOXO1 cascade and the conserved phosphorylation site of FOXO1 by MST1. Source data are provided as a Source Data file

To reveal the factors that activate MST1 during sprouting angiogenesis, we analyzed the publicly available microarray data (GSE19284) that identified enriched genes in tip ECs^39^. Of note, the genes related to ROS, including ROS biosynthesis and response to ROS, and those related to hypoxia were enriched in tip ECs compared to non-ECs (Fig. 5a). These findings led us to hypothesize that a hypoxic microenvironment in the vascular front constantly induces intracellular ROS synthesis, which activates the MST1–FOXO1 cascade at tip ECs. To address this hypothesis, primary cultured human umbilical vascular endothelial cells (HUVECs) were exposed to hypoxia (1% O_2_). After hypoxia exposure, increased MST1 phosphorylation at Thr183 (pMST1) was detected already at 30 min, gradually increased without alteration of the activity of canonical Hippo pathway, peaked at 6 h, decreased thereafter, and returned to basal level at 36 h. Under the same condition, increased changes in HIF1α protein level and FOXO1 phosphorylation at Ser212 (pFOXO1) were positively correlated with increases in pMST1 (Fig. 5b, c; Supplementary Figs. 6 and 7a–e). These data imply that hypoxia activates MST1 and FOXO1 in ECs, leading us to ask whether ROS directly contributes to activate the MST1–FOXO1 cascade and where ROS is produced in ECs under hypoxia.

The process of ROS biosynthesis by NADPH oxidase, xanthine oxidase and mitochondrial electron transport chain requires oxygen as a substrate. However, even though the exact mechanism remains elusive, ROS can be produced in hypoxia, which is mainly dependent on mitochondrial electron transport chain^40,41^. To delineate the relationship among hypoxia-intracellular ROS–MST1–FOXO1 in ECs, we treated Trolox (a ROS scavenger) and rotenone (a mitochondrial electron transport chain I inhibitor) to the HUVECs. Both Trolox and rotenone suppressed the hypoxia-induced stabilization of HIF1α protein level by 31 and 48% and pMST1 by 35 and 41%, respectively (Fig. 5d, e), indicating that hypoxia-induced MST1 activation is primarily mediated through intracellular ROS biosynthesis at the mitochondria in ECs. We then asked whether pFOXO1 depends on pMST1, according to previous report^19,22^. Of note, immunoprecipitation analysis using the HUVECs revealed that endogenous MST1 physically interacts with endogenous FOXO1 (Fig. 5f). Moreover, depletion of MST1 using its corresponding small interfering RNA (siRNA) abrogated the hypoxia-induced pFOXO1 in parallel (Fig. 5g). These results imply that MST1 directly mediates pFOXO1 through hypoxia-induced intracellular ROS biosynthesis (Fig. 5h), which is consistent with previous findings in other cell types^19^.

### MST1 governs the nuclear import of FOXO1 under hypoxia

**Fig. 6:**
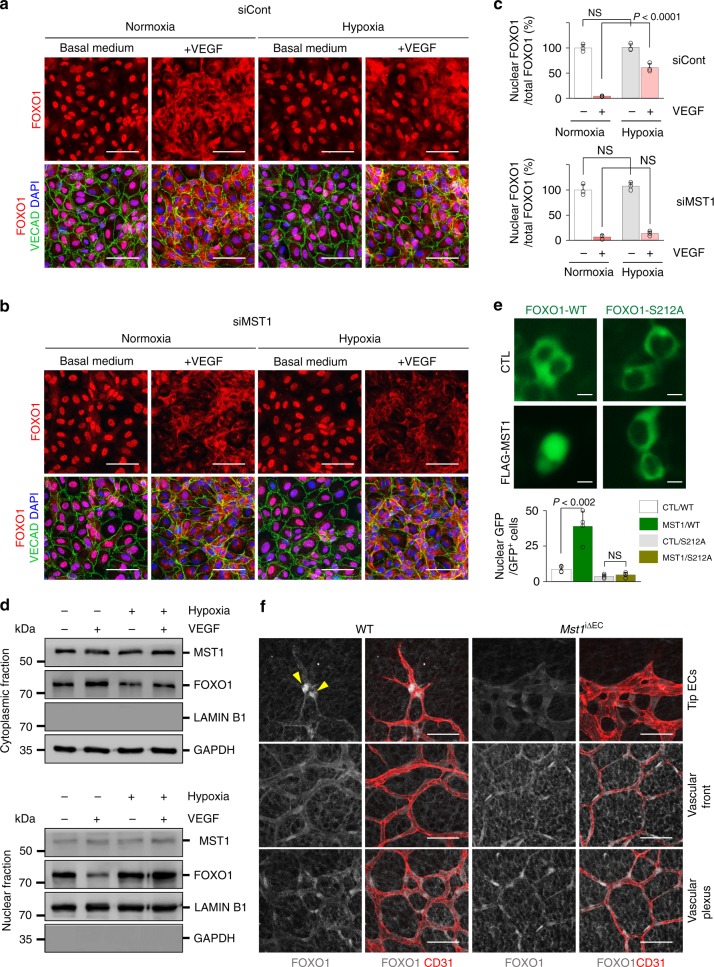
MST1 activation governs to promote nuclear import of FOXO1 under hypoxia. **a**–**c** Images and comparisons of the nuclear enrichment of FOXO1 in siCont-ECs and siMST1-ECs exposed to normoxia or hypoxia (1% O_2_) in the absence (−) or presence (+) of VEGF (200 ng/ml) for 30 min (*n* = 3, each group). Scale bars, 20 μm. Data represent mean (bar) ± s.d. (error bars). *P* values, normoxia with VEGF versus hypoxia with VEGF by one-way ANOVA with Tukey’s post hoc test. NS not significant. **d** Immunoblot analyses of indicated proteins in nuclear and cytoplasmic fractions of HUVECs exposed to normoxia (−) or hypoxia (1% O_2_) (+) without (−) or with (+ ) VEGF stimulation (200 ng/ml, 30 min). **e** Images and comparisons of the nuclear enrichment of GFP in HEK293T cells transfected with gene constructs encoding either GFP-tagged FOXO1 (FOXO1-WT or WT) or non-phosphorylatable FOXO1 (FOXO1-S212A or S212A) together with either control vector (CTL) or gene construct encoding MST1 (FLAG-MST1 or MST1) [*n* = 161(CTL/WT), 164(MST1/WT), 151(CTL/S212A), 154(MST1/S212A)]. Scale bars, 10 μm. Data represent mean (bar) ± s.d. (error bars). *P* values, CTL/WT versus MST1/WT or CTL/S212A versus MST1/S212A by one-way ANOVA with Tukey’s post hoc test. NS not significant. **f** Images of subcellular localizations of FOXO1 in CD31^+^ retinal vessels of WT and *Mst1*^i∆EC^ mice at P6. Note that the nuclear enriched FOXO1 at tip ECs (yellow arrowheads) is impaired in *Mst1*^i∆EC^ mice, while the distributions of FOXO1 at vascular front and plexus are unaltered. Scale bars, 50 μm. Source data are provided as a Source Data file

Considering activation of phosphatidylinositol-3-OH kinase (PI3K)/AKT signaling by VEGF promotes nuclear export of FOXO1 through phosphorylation at Ser256, we postulated that activated MST1 may predominantly promote nuclear import of FOXO1 through phosphorylation at Ser212 against the export stimulated by VEGF-induced PI3K/AKT activation. To decipher this postulation, we confirmed that hypoxia–MST1–FOXO1 cascade operates independently of VEGF/VEGFR2-PI3K-AKT-FOXO1 cascade (Supplementary Fig. 7 f, g). Then, the HUVECs transfected with the siRNA for control (siCont-ECs) or the siRNA for *MST1* gene (siMST1-ECs) were stimulated with VEGF (200 ng/ml) for 30 min under normoxia or hypoxia (1% O_2_). Under normoxia, FOXO1 was mainly localized in nuclei of siCont-ECs but mostly was (96%) exported to cytoplasm upon VEGF stimulation. This VEGF-stimulated dramatic FOXO1 shuttling was largely (58%) abrogated under hypoxia. In contrast, this shuttling was consistently muted in siMST1-ECs under both normoxia and hypoxia (Fig. 6a–c). Immunoblot analysis following nuclear–cytoplasmic fractionation of the HUVECs confirmed that MST1 was stably settled in the cytoplasm regardless of hypoxia and VEGF stimulation, whereas FOXO1 was dynamically shuttled between cytoplasm and nucleus in response to VEGF under normoxia but not under hypoxia (Fig. 6d). To confirm the effect of MST1 and phosphorylation of FOXO1 at Ser212 in determining the subcellular localization of FOXO1, we transfected HEK293T cells with gene constructs encoding either GFP-tagged FOXO1 (GFP-FOXO1-WT) or non-phosphorylatable FOXO1 (GFP-FOXO1-S212A) together with either control vector (CTL) or gene construct encoding MST1 (FLAG-MST1). MST1 induced the nuclear import of FOXO1 in GFP-FOXO1-WT-transfected cells (8.8% in CTL transfected cells compared with 38.9% in FLAG-MST1 transfected cells of all GFP-expressing cells), while it was markedly reduced in GFP-FOXO1-S212A-transfected cells (3.7% in CTL transfected cells compared with 4.7% in FLAG-MST1 transfected cells of all GFP-expressing cells) (Fig. 6e). These results indicate that the phosphorylation of FOXO1 at Ser212 by MST1 mediates hypoxia-induced nuclear import of FOXO1.

Consistent with these in vitro findings, compared to those of WT mice at P6, distinctly nuclear localized FOXO1 largely disappeared in tip ECs of growing retinal vessels of *Mst1*^i∆EC^ mice. In contrast, no apparent differences were detected in the localizations of FOXO1 in the ECs of the vascular front and plexus. These results imply that MST1 activation plays a dominant role in nuclear–cytoplasmic shuttling of FOXO1 in tip ECs that are exposed to extreme hypoxia (Fig. 6f).

### MST1–FOXO1 cascade establishes cell polarity in EC migration

**Fig. 7:**
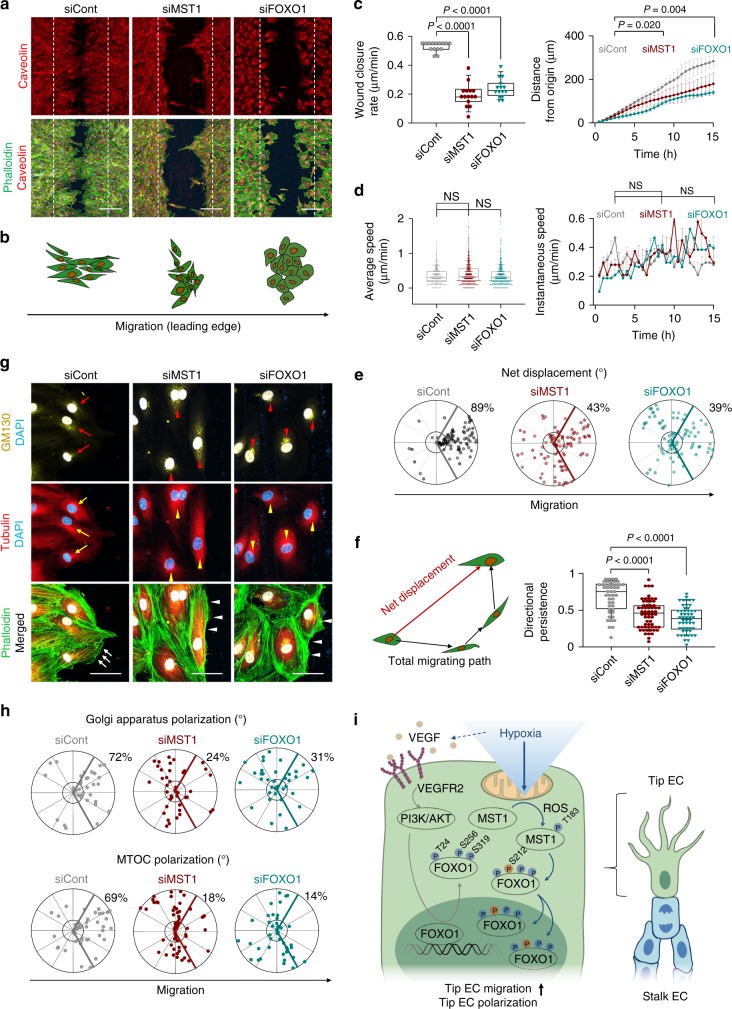
MST1–FOXO1 cascade establishes cell polarity in EC migration. **a** Images of phalloidin^+^ actin cytoskeleton and caveolin in indicated ECs subjected to the wound scratch. The dashed lines indicate the initial margin of wound scratch. Scale bars, 200 μm. **b** Schematic pictures depicting cell morphology at the leading edge in indicated ECs. **c** Comparisons of indicated parameters in indicated ECs. *n* = 15, each group in the left panel. *n* = 10–15, each group at each time in the right panel. **d** Comparisons of indicated parameters in indicated ECs. *n* = 1941(siCont), 2190(siMST1), 2217(siFOXO1) in the left panel. *n* = 24–25, each group in the right panel. **e** Polar plots showing net displacement. *n* = 94(siCont), 98(siMST1), 88(siFOXO1). **f** Schematic picture depicting net displacement and total migrating path and comparison of single cell directional persistence defined by net displacement divided by total migrating path length in indicated ECs. *n* = 47(siCont), 61(siMST1), 52(siFOXO1). **g** Images of phalloidin^+^ actin cytoskeleton, α-tubulin^+^ microtubule, GM130^+^ Golgi apparatus and DAPI in the leading edge of indicated ECs at 9 h after initiating cell migration. Note that Golgi apparatus and microtubule (red and yellow arrows) in siCont-ECs are localized in the direction of cell migration, while those (red and yellow arrowheads) in siMST1-ECs and siFOXO1-ECs are localized randomly. Moreover, siMST1-ECs and siFOXO1-ECs rarely show lamellipodia (white arrowheads) compared to siCont-ECs (white arrows). Scale bars, 50 μm. **h** Polar plots showing Golgi apparatus [*n* = 36(siCont), 41(siMST1), 38(siFOXO1)] and microtubule organizing centre (MTOC) polarization [*n* = 45(siCont), 57(siMST1), 41(siFOXO1)]. **i** Schematic picture summarizing predominant role of MST1 in the nuclear import of FOXO1 against VEGF/VEGFR2-PI3K/AKT pathway at tip ECs. **c**, **d**, **f** Box plots represent Center line, median; Box limits, upper and lower quartiles; whiskers, s.d. Right panel in **c** represents mean (points) ± s.d. (error bars). Right panel in **d** represents Bar, mean; Points, median. *P* values, versus siCont by one-way ANOVA with Tukey’s post hoc test. NS not significant. **e**, **h** the bold lines indicate 120° region centered on the vector which is vertical to the wound scratch direction. The numbers indicate the frequency of dots within the 120° region of the bold line. Source data are provided as a Source Data file

Given that *Mst1*^i∆EC^ and *Foxo1*^i∆EC^ mice failed to induce tip EC polarization, we postulated that the MST1–FOXO1 cascade is critical for inducing tip EC polarization into the avascular area. To evaluate the role of the MST1–FOXO1 cascade in establishing cell polarity during directional EC migration into the avascular area, we employed a wound scratch assay in confluent siCont-ECs, siMST1-ECs, and siFOXO1-ECs (HUVECs transfected with siRNA targeting the *FOXO1* gene) and examined the EC behaviors at the wound margin (Supplementary Fig. 8a, b). While the gap was 95% closed in siCont-ECs at 15 h after scratching, it was only 35 and 43% closed in siMST1-ECs and siFOXO1-ECs, respectively. Of importance, siMST1-ECs and siFOXO1-ECs lost polarization toward the gap area in the leading edge, while siCont-ECs were polarized toward the gap. (Fig. 7a–c and Supplementary Movies 1–3).

We further tracked each EC using a CellTracker^42^ and analyzed speed and directionality of the ECs. siCont-ECs showed a linear cell morphology and migrated toward the gap. siMST1-ECs showed a mixed cell morphology of linear and cobblestone-like appearance and migrated randomly regardless of the direction of the gap, and siFOXO1-ECs exhibited a cobblestone-like appearance with small displacement between imaging intervals (Supplementary Fig. 8c and Movies 4–6). Although the average and instantaneous speeds of each EC among siCont-, siMST1-, and siFOXO1-ECs were similar for 15 h, ∼90% of siCont-ECs were displaced into the gap, while 57 and 61% of siMST1- and siFOXO1-ECs, respectively, were randomly displaced (Fig. 7d, e). Moreover, while siCont-ECs exhibited persistent and directional migration, siMST1- and siFOXO1-ECs randomly migrated without directional persistence (defined by the net displacement divided by the total migrating path of each cell) (Fig. 7f). Furthermore, at 9 h after scratching, in the leading edge of siMST1- and siFOXO1-ECs compared with those of siCont-ECs, there were randomly positioned Golgi apparatus, microtubule organizing centers including microtubules (shown by tubulin) and centrosome (shown by pericentrin), and caveolin; fewer incorporated vinculin into the focal adhesion site; and smaller lamellipodia (Fig. 7g, h and Supplementary Fig. 8d–g). These results indicate that MST1 and FOXO1 are crucial for establishing endothelial polarity in the direction of cell migration.

### MST1–FOXO1 commonly regulates genes related to EC migration

To elucidate how MST1 and FOXO1 regulate directional EC migration at the transcriptional level, we performed RNA sequencing analysis in siCont-, siMST1-, and siFOXO1-ECs. Ingenuity Pathway Analysis was used to achieve Gene Ontology (GO) analysis in comparisons of siCont-ECs versus siMST1-ECs and siCont-ECs versus siFOXO1-ECs. The GO term Cellular Movements was ranked first and second in the comparison of siMST1-ECs and siFOXO1-ECs to siCont-ECs, respectively (Supplementary Fig. 9a), implying that MST1 and FOXO1 commonly regulate genes related to Cellular Movements. To decipher how much MST1 and FOXO1 cooperate to regulate genes related to cellular movements, we employed the EdgeR method in R and found 659 and 3,680 differential expressed genes (DEGs) in the comparisons of siCont-ECs versus siMST1-ECs and siCont-ECs versus siFOXO1-ECs, respectively. Only 369 genes among DEGs were commonly regulated by MST1 and FOXO1 (Supplementary Fig. 9b). Furthermore, we clustered the commonly regulated 369 genes and found that 286 genes were commonly up- or down- regulated by MST1 and FOXO1 (Supplementary Fig. 9c), which were assumed to be the genes regulated by the MST1–FOXO1 cascade. Of interest, GO biological process analysis with the genes regulated by MST1–FOXO1 cascade revealed that GO terms including EC migration, sprouting angiogenesis, and cell-substrate junction assembly were ranked in the top 10 enrichment candidates, and GO terms related to angiogenesis showed significantly high Q scores [-log_10_(*p*-value)] (Supplementary Fig. 9d, e). Collectively, these results delineate that MST1–FOXO1 cascade establishes endothelial cell polarity through transcriptional control of cell migration and adhesion which affects cell polarity and vice versa^43^ (Fig. 7i).

Furthermore, to identify the target genes of MST1–FOXO1 cascade, we transfected each gene constructs; GFP-FOXO1-WT, GFP-FOXO1-S212A, or CTL into the HUVECs. Intriguingly, *CCBE1, CXCL8, RSPO3*, and *FGF2* were upregulated in GFP-FOXO1-WT compared with CTL. Conversely, *CCBE1* and *CXCL8* were downregulated in GFP-FOXO1-S212A compared with GFP-FOXO1-WT. In addition, when MST1 was depleted in the HUVECs, *CCBE1* and *CXCL8* were downregulated; however, this was reversed when the HUVECs were also transfected with GFP-FOXO1-WT. Taken together, MST1-FOXO1 cascade regulates the expressions of *CCBE1* and *CXCL8* in a pFOXO1-dependent manner in the HUVECs (Supplementary Fig. 10a–d).

### MST1–FOXO1 cascade is required for pathological angiogenesis

**Fig. 8:**
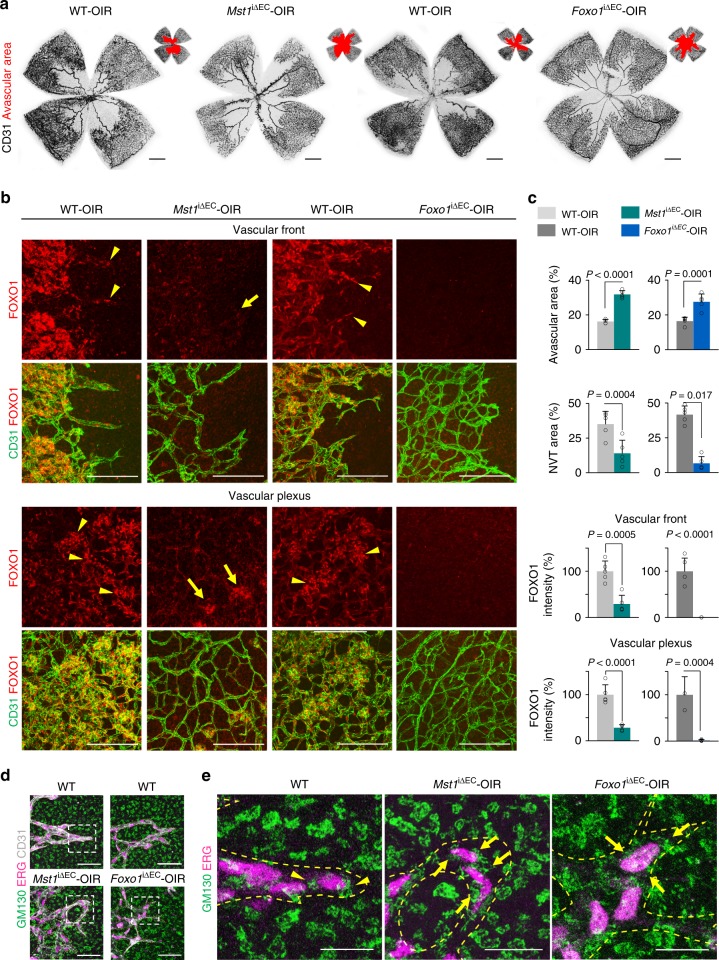
MST1–FOXO1 cascade is required for pathologic angiogenesis. **a** Images of CD31^+^ vessels in the superficial layer of retinas and avascular area (red) in WT-OIR, *Mst1*^i∆EC^-OIR, and *Foxo1*^i∆EC^-OIR mice. Scale bars, 500 μm. **b** Images of subcellular localization of FOXO1 in CD31^+^ vessels at vascular front (revascularization) and vascular plexus (neovascularization) in WT-OIR, *Mst1*^i∆EC^-OIR, and *Foxo1*^i∆EC^-OIR mice. Scale bars, 100 μm. Note that WT-OIR mice exhibited a nuclear localization of FOXO1 (yellow arrowheads), while *Mst1*^i∆EC^-OIR mice showed a diffuse nucleocytoplasmic localization of FOXO1 (yellow arrows) in tip ECs and NVT ECs. **c** Comparisons of indicated parameters in WT-OIR (*n* = 5), *Mst1*^i∆EC^-OIR (*n* = 5) and *Foxo1*^i∆EC^-OIR (*n* = 5) mice. Data represent mean (bar) ± s.d. (error bars). *P* values, versus WT by two-tailed unpaired *t*-test. **d** Images of CD31^+^ vessels, ERG^+^ nuclei of ECs and GM130^+^ Golgi apparatus at tip ECs in WT-OIR, *Mst1*^i∆EC^-OIR and *Foxo1*^i∆EC^-OIR mice. The images of the inset (white dashed-line boxed) are magnified in **e**. The yellow dashed line outlines CD31^+^ vessels. Scale bars, 50 μm. **e** Images of ERG^+^ nuclei of ECs and GM130^+^ Golgi apparatus at tip ECs in WT-OIR, *Mst1*^i∆EC^-OIR, and *Foxo1*^i∆EC^-OIR mice. The yellow dashed line outlines CD31^+^ vessels. Note that GM130^+^ Golgi apparatus are polarized towards the anterior or posterior of the nuclei in tip ECs of WT-OIR mice (yellow arrowheads), while such polarization is lost in tip ECs of *Mst1*^i∆EC^-OIR and *Foxo1*^i∆EC^-OIR mice (yellow arrows). Scale bars, 100 μm. Source data are provided as a Source Data file

To examine whether endothelial MST1 is required to maintain retinal vessel integrity, we deleted MST1 in ECs of *Mst1*^i∆EC^ mice from age 8 weeks and analyzed them 4 weeks later (Supplementary Fig. 11a). No definite differences were found between WT and *Mst1*^i∆EC^ mice in radial length, vascular density, RBC leakage, and distributions of VE-cadherin and ERG^+^ nuclei of the retinal vessels (Supplementary Fig. 11b–e). These results indicate that endothelial MST1 is dispensable for maintaining integrity of retinal vessels. To see whether endothelial MST1 and FOXO1 has a significant role in pathologic angiogenesis, we employed an oxygen-induced retinopathy (OIR) model (Supplementary Fig. 11f). *Mst1*^i∆EC^-OIR and *Foxo1*^i∆EC^-OIR mice exhibited impaired revascularization (196 and 167% increased avascular area, respectively) and neovascularization [60 and 85% reduced neovascular tuft (NVT) area, respectively] compared with WT-OIR mice (Fig. 8a, c). FOXO1 was enriched in the nuclei of ECs and Angpt2 was highly expressed in tip ECs and NVT ECs, where aberrant sprouting occurs, compared with adjacent ECs in WT-OIR mice. However, *Mst1*^i∆EC^-OIR mice showed 70 and 71% reduced FOXO1 intensity and 64 and 71% reduced Angpt2 expression in tip ECs and NVT ECs, respectively, compared with those of WT-OIR mice. Furthermore, Angpt2 expression was nearly absent in tip ECs and NVT ECs of *Foxo1*^i∆EC^-OIR mice compared with those of WT-OIR mice (Fig. 8b, c and Supplementary Fig. 11g, h). In addition, the Golgi apparatus were polarized towards the anterior or posterior of the nuclei in tip ECs of WT-OIR mice. On the contrary, such polarizations were lost in tip ECs of *Mst1*^i∆EC^-OIR and *Foxo1*^i∆EC^-OIR mice (Fig. 8d, e). Thus, these results imply that MST1 facilitates the nuclear import of FOXO1 in tip ECs as well as in NVT ECs during OIR progression. Moreover, endothelial MST1-FOXO1 cascade is required for revascularization and neovascularization and contributes to establishing endothelial polarity in revascularization.

## Discussion

Rapidly growing tissues during development and solid tumors swiftly expanding into surrounding parenchymal tissues constantly face hypoxia due to inadequate angiogenesis and blood supply^44,45^. In turn, hypoxia is a leading driver for sprouting and intussusceptive angiogenesis, but how it guides tip ECs toward the hypoxic area has been an enigma. An ample number of previous studies^1,46–49^ have suggested that the VEGF gradient could be a major determinant for tip EC polarization during sprouting angiogenesis. However, there is devoid of sprouting angiogenesis with accompanying tip EC polarization in VEGF-rich adult organs such as endocrine glands, liver, kidney, and muscles^50^, raising the possibility that hypoxia could activate an alternative pathway for guiding tip ECs in sprouting angiogenesis into the avascular region. In this study, we uncovered a coupling between extracellular hypoxia and intracellular ROS–MST1–FOXO1 pathway, which establishes the polarity of tip EC during sprouting angiogenesis.

Among diverse roles of MST1, we have defined the roles in the behavior of tip ECs of growing blood vessels. Of note, our findings indicate that hypoxia-induced ROS biosynthesis from mitochondria could be a major upstream regulator of MST1 activation in ECs. Activated MST1 promotes planar and perpendicular vascular branching for regulating tip EC polarity and sprouting angiogenesis. In general, MST1 can be activated in two ways: cleavage and phosphorylation^26^. Cleaved MST1 under apoptotic stimuli translocates from cytosol into the nucleus, promoting chromatin condensation during cellular apoptosis^26^. On the other hand, a full-length MST1 can be autophosphorylated at Thr183 under cellular stresses by disrupting the interaction between thioredoxin-1 and SARAH (Salvador/RASSF1/Hippo) domain^51^, and activated MST1 phosphorylates cytosolic substrates such as SAV, LATS1/2, and FOXOs^26^. Not surprisingly, the physiologic activity of MST1 and its cellular substrates are cell type- and context-dependent^20,52,53^. Our analyses using the genetically modified mice revealed that endothelial MST1 does not undertake a canonical Hippo pathway. Instead, endothelial MST1 phosphorylates FOXO1 under hypoxia in HUVECs, and this kinase–substrate interaction drives nuclear import of FOXO1 in tip ECs. Of importance, this hypoxia–MST1–FOXO1 cascade operates independently of VEGF/VEGFR2 signaling in regulating sprouting angiogenesis. Thus, endothelial MST1 is involved in the regulatory cascade for nuclear transport of FOXO1 rather than its cleavage and in regulation of apoptosis and chromatin condensation or its phosphorylation to LATS1/2 for the canonical Hippo pathway for establishing tip EC polarity during sprouting angiogenesis.

Then, how does nuclear FOXO1 regulates polarity of tip ECs? Beyond the traditional roles of FOXOs as transcriptional factors for maintaining cellular homeostasis^54^, the newly identified roles of FOXOs in neuronal polarization and positioning have been highlighted^55,56^. They control axon–dendrite polarity and morphogenesis in developing granular neurons through regulating polarity-associated gene expression^56^. Thus, these novel roles of FOXOs in neurons could be shared with the roles described here for FOXO1 in tip EC polarization in terms of cell behaviors and regulating polarity-associated gene expression. Nevertheless, this involvement is apart from the deciphered role of FOXO1 in metabolic gatekeeping to maintain EC homeostasis^11^ during establishment of the vascular plexus of growing vessels. In this regard, determining specific FOXO1 transcriptional output will be of interest.

Several models have been proposed to explain the specific transcriptional regulation of FOXO1^54^. FOXO1 can bind numerous sites with members of transcriptional machinery because the length of its DNA binding motif is short^57^, on the other hand, FOXO1 interacts with other transcriptional factors or cofactors and can initiate transcriptional activity with or even without direct binding to DNA^58^. Of particular note, FOXO1 that is phosphorylated by MST1 could not bind to DNA, even if it translocates to the nucleus^59^. Together, our finding suggest that MST1–FOXO1 cascade may regulate polarity/migration-associated genes by interacting with other transcriptional machineries rather than direct binding to DNA, which needs further investigations. In addition, although we identified the hypoxia-intracellular ROS-MST1 as the upstream signal of FOXO1 at tip ECs, a limitation of this study is the inability to analyze the regulatory roles of FOXO1 in gene expression and PTMs of FOXO1 in ECs in different regions of growing retinal vessels. Defined analysis at the single cell level at the different regions of ECs is warranted for further study to understand a context-dependent role of FOXO1 during angiogenesis.

In conclusion, this study demarcates a crucial coupling between extracellular hypoxia and intracellular ROS–MST1–FOXO1 pathway, which establishes the polarity of tip ECs during developmental and pathologic angiogenesis.

## Methods

### Mice and treatment

Specific pathogen-free (SPF) C57BL/6 J mice and R26-tdTomato were purchased from the Jackson Laboratory. *VE-cadherin*-Cre-ER^T2 28^, *Mst1*^flox/flox^^27^, *Mst2* null^34^, and *Foxo1*^flox/flox^^38^ mice were transferred, established and bred in SPF animal facilities at KAIST and fed with free access to a standard diet (PMI Lab diet) and water. In order to induce Cre activity in the Cre-ER^T2^ mice, tamoxifen (Sigma-Aldrich, T5648) was given with following dosages and schedules: for neonatal mice, 100 μg of tamoxifen dissolved in corn oil (Sigma-Aldrich, C8267) was injected into the stomach daily from P1 to P3; for OIR mice, 200 μg of tamoxifen was injected intraperitoneally (i.p.) daily from P12 to P14; for adult mice aged over 8 weeks, 2 mg of tamoxifen was injected i.p. for 5 consecutive days from the indicated time point. For anesthesia, mice were injected i.p. with the anesthetic solution (ketamine 40 mg/kg and xylazine 5 mg/kg). We complied with all ethical regulations for animal testing and research and performed all animal experiments under the approval from the Institute Animal Care and Use Committee (No. KA2017-31) of KAIST.

### Histological analyses

Briefly, eyeballs were enucleated and fixed in 4% paraformaldehyde (PFA) for 20 min at room temperature (RT). After preparing the retinas from eyeballs, they were additionally fixed in 1% PFA for 1 h at RT. Samples were blocked with 5% donkey (or goat) serum in PBST (0.3% Triton X-100 in PBS) for 30 min, incubated in primary antibodies (diluted at a ratio of 1:200 in blocking solution) at 4 °C overnight, washed in PBST, and incubated in secondary antibodies in blocking solution at RT for 2 h. The retinas were washed in PBST and mounted on microscope glass slides with Vectashield (Vector Laboratories, H-1200). IF staining of brain were performed as follows. Mice were anesthetized and perfusion-fixed with 4% PFA. After dissecting the brain from skull, the brain was additionally fixed in 4% PFA at 4 °C for 6 h. The samples were cut into 150 μm sections by a vibratome (Leica, VT1200 S), immunostained, and mounted. Primary cultured HUVECs were fixed in 2% PFA for 10 min at RT. After blocking, the cells were stained with primary and secondary antibodies using the same procedure as tissue staining, and mounted with DAKO mounting medium. Primary antibodies and reagent used for IF were as follows: hamster anti-CD31 monoclonal (Millipore, MAB1398Z); isolectin B4 (IB4), Alexa Fluor 594-conjugated (Thermo Fisher Scientific, I21413); rat anti-VE-cadherin monoclonal (BD Pharmingen, 555289); mouse anti-VE-cadherin monoclonal (BD Biosciences, #555661); rabbit anti-ERG monoclonal (Abcam, ab92513); rabbit anti-cleaved-caspase-3 (Cl-CASP3) polyclonal (CST, #9664); rabbit anti-pHH3 polyclonal (Millipore, 05-806); rabbit anti-collagen IV polyclonal (Bio-Rad, 2150-1470); rat anti-ICAM2 monoclonal (BD Pharmingen, 553326); rat anti-TER119 monoclonal (BD Pharmingen, 561033); rabbit anti-GLUT1 polyclonal (EMD Millipore, 07-1401); mouse anti-GM130 monoclonal, Alexa Fluor 647-conjugated (BD Biosciences, #558712); rabbit anti-TAZ polyclonal (Sigma-Aldrich, HPA007415); rabbit anti-FOXO1 monoclonal (CST, #2880); human anti-Angpt2 monoclonal (clone 4H10)^60^; Phalloidin, Alexa Fluor 488- conjugated (Thermo Fisher Scientific, A12379); Phalloidin, Alexa Fluor 594-conjugated (Thermo Fisher Scientific, A12381); mouse anti-caveolin-1 monoclonal (Abcam, ab17052); rabbit anti-GM130 polyclonal, Alexa Fluor 555-conjugated (Thermo Fisher Scientific, PA1-077-A555); rabbit anti-GM130 polyclonal, Alexa Fluor 647-conjucated (Thermo Fisher Scientific, PA1-077-A647); mouse anti-alpha tubulin monoclonal, Alexa Fluor 488-conjugated (Thermo Fisher Scientific, #322588); mouse anti-vinculin monoclonal, Alexa Fluor 488-conjugated (Thermo Fisher Scientific, #53-9777-80); mouse anti-pericentrin monoclonal (Abcam, ab28144). Secondary antibodies (diluted at a ratio of 1:1000 in PBST) include: FITC-, Cy3-, Cy5-conjugated anti-hamster IgG, anti-rat IgG, anti-rabbit IgG, anti-human IgG, and anti-goat IgG (Jackson ImmunoResearch). Images of all samples were obtained using a confocal microscope (Zeiss, LSM880) and processed with imaging softwares, ZEN (Zeiss) and Adobe Photoshop (Adobe).

### EdU incorporation assay for proliferating ECs

Six milligrams of 5-ethynyl-2′-deoxyuridine (EdU) (Invitrogen, A10044) was dissolved in 1 ml of Milli-Q water as a stock solution. Then, 5 μl of the stock solution per gram of body weight was injected i.p. 3 h before killing. Retinas were isolated and processed as described above. EdU-incorporated cells were detected with the Click-iT EdU Alexa Fluor-555 Imaging Kit (Invitrogen, C10338).

### Cell culture and treatment

Pooled primary cultured HUVEC cells were purchased from Lonza (C2519A). HUVECs were cultured in EGM2 media (Lonza) in culture dishes coated with 0.1% gelatin. HUVECs at passages 3–6 were used for this study. HUVECs were incubated under hypoxia (1% O_2_) for indicated times. VEGF (200 ng/ml, 293-VE-010, R&D systems), Rotenone (2.5 μM, R8875, Sigma-Aldrich) and Trolox (1 mM, 648471, Millipore) were treated to HUVECs. HEK 293 T (human embryonic kidney) cells were cultured in Dulbecco’s modified Eagle’s medium (Welgene, LM001-05). Cells were maintained at 37 °C, 5% CO_2_ and 95% relative humidity.

### RNA interference

HUVECs were transfected with a pool of siRNAs using Lipofectamine RNAiMAX (Invitrogen) according to the manufacturer’s protocols. The following target sequences were used: human MST1 (5′- GGGCACTGTCCGAGTAGCAGC-3′), human FOXO1 (Bioneer, 1058762) and human FOXO3 (Bioneer, 2309-1). GL2 siRNA (5′- CGTACGCGGAATACTTCGA-3′) was used as negative control. The HUVECs were harvested 48–72 h after transfection.

### Retroviral infection

The GFP-tagged human FOXO1 cDNA was cloned into the pMSCV-puro vector (GFP-FOXO1-WT). Non-phosphorylatable FOXO1 (GFP-FOXO1-S212A) was generated by overlap extension PCR. The FLAG-tagged human MST1 cDNA was cloned into the pMSCV-puro vector (FLAG-MST1). The retroviruses were produced in HEK 293 T cells with each indicated gene constructs using Lipofectamine P3000 (Invitrogen) and HUVECs were infected with each indicated retrovirus using Hexadimethrine bromide (Sigma-Aldrich). pMSCV-puro vector was used as a control vector.

### Wound scratch assay

Briefly, wound scratch assay were performed on confluent layers of transfected HUVECs. 48 h after siRNA transfection, cells were seeded into 24-well cell culture plate confluently and were incubated for 24 h to allow them to adhere. Migration was initiated by scratching wound with the 200 μl pipet tip followed by two washes with EGM2 media. Phase images of migration were taken every 10 min for 15 h on a Cell observer (Zeiss). For IF analyses of cell migration, cells were maintained on 8-well glass slide (Lab-Tek, 154534) and fixed with 2% PFA for 10 min at 9 h after initiating cell migration by scratch. Cells were maintained in an incubation chamber at 37 °C, 5% CO_2_ and 95% relative humidity during the experiment.

### Morphometric analyses

Morphometric measurements of retinas and brains were performed by using the ImageJ software (NIH). Radial length of retinal vessel was measured as the distance from the optic disc to the peripheral vascular front in each leaflet of the retina and averaged. Retinal vascular density was measured as CD31^+^ or IB4^+^ retinal vessel area divided by total measured area of the retina and presented as a percentage. The number of branching points was measured manually in four 500 µm × 500 µm fields located between an artery and a vein in each retina and averaged. The number of ERG^+^ nuclei of ECs was counted in five 200 µm × 200 µm fields and averaged per sample. The number of EdU incorporated ECs, pHH3^+^ labeled proliferating ECs and CASP3^+^ apoptotic ECs were measured in four 500 µm × 500 µm fields and averaged per sample. Perpendicular growth of retinal vessels was measured as the length of the vessels reaching out from the superficial layer to the vascular front ahead to the deep layer. The total number of sprouts and filopodia was first measured in a 500 µm × 500 µm field and then normalized to a length of 100 µm along the angiogenic front, which was measured four times per sample and averaged. The number of filopodia per sprout was calculated by dividing the number of filopodia by the number of sprouts, which was measured in five sprouts per sample and averaged. Sprout length was examined in six 100 µm × 100 µm areas of vascular front in each retina and averaged. The angle of ERG^+^ nuclei of ECs was measured as the angle of the nuclear long axis, which was projected in the first quadrant with 90° being considered as the direction of filopodia extension. Nuclear ellipticity was calculated by the distance of nuclear long axis (Height) divided by the maximum vertical distance to the nuclear long axis (Width). RBC leakage was measured in 200 µm × 200 µm fields as RBC-stained area outside of the vessels divided by the measured area and averaged. NVT area in the OIR model was measured using the Lasso tool of Adobe Photoshop software and divided by total CD31^+^ vessel area. Staining intensities were measured in ten regions of interest (ROIs) of vessels or, if indicated, other vascular regions in each retina and averaged. For comparison, the values were subtracted by the background signals in non-vascularized areas and averaged, which were then normalized by the average of those of control and presented as fold change or percentage. The stained area of specific molecules in retinal vessels was measured using the same threshold values in ImageJ software and divided by the total area of CD31^+^ or IB4^+^ retinal vessels. High-resolution confocal images were taken using ×40 lens and then analyzed using the IMARIS software (Bitplane). Analyses of the 3D structures of ERG^+^ nuclei of ECs and CD31^+^ vessels were performed using the surface module of IMARIS software (Bitplane). Vascular density in brains was measured as CD31^+^ vessel area divided by total measured area of brain with Z-projected images of 150 μm-thickness and presented as a percentage. Identical measurements of those performed with retina were employed for other morphometric analyses for brain. Nuclear FOXO1 expression in HUVECs was measured using ImageJ software as the co-localized FOXO1^+^ and DAPI^+^ area divided by total FOXO1^+^ area. For cell migration and polarity analyses, the width of the wounds was analyzed using ImageJ software and wound closure rate (μm/min) was calculated by dividing the difference between the width at 0 h and 15 h by 900 (15 h × 60 min) in six measurements per sample. The Golgi apparatus, MTOC and pericentrin polarization were measured in 15–20 cells per sample using ImageJ software as the angle obtained from the centre of the nucleus to the organelles.

### Analyses for cell migration characteristics

The cell migration characteristics were analyzed using CellTracker^42^ implemented in MATLAB (MATLAB R2017a, MathWorks). Three images or videos were taken for each sample and three replicates were analyzed for siCont-, siMST1- and siFOXO1-ECs. In each image or video, 18–20 cells were tracked. Raw data from CellTracker were extracted in pixel unit which were converted into μm (1784.22 μm/1920 pixel = 0.93 μm/pixel). Average speed and net displacement were compared among each groups from the extracted data. Distance from origin and instantaneous speed were calculated and compared from the data measured every 30 min. Directional persistence was calculated as the net displacement divided by the total migrating path of each cell and compared among each groups.

### Generation of the anti-phospho-FOXO1 (Ser212) polyclonal antibody

The rabbit antibody against phospho-FOXO1 (Ser212) was generated as follows^13,19^. Briefly, the phosphopeptide antigen C-SAGWKNpSIRHNLS was synthesized, HPLC purified, conjugated to keyhole limpet hemocyanine, and injected into two rabbits over an 8-week period (one primary injection and three boosting injections) (Abclon). The antiserum was obtained for affinity purification to remove non-specific antibodies, increase sensitivity and reduce background.

### Immunoblotting and immunoprecipitation assays

The cells were lysed on ice in RIPA lysis buffer supplemented with protease and phosphatase inhibitors (CST, #5872). Cell lysates were centrifuged for 10 min at 4 °C, 15,000 *g*. Protein concentrations of the supernatants were quantitated using the detergent-insensitive Pierce BCA protein assay kit (Thermo Scientific, 23227). Lamni buffer was added to total protein lysates and samples were denatured at 95 °C for 5 min. Aliquots of each protein lysate (10–20 μg) were subjected to SDS–polyacrylamide gel electrophoresis.

For immunoprecipitation analysis, cells were lysed on ice in NETN buffer (20 mM Tris-HCl [pH 7.4], 100 mM NaCl, 1 mM EDTA, 0.5% NP-40) with protease and phosphatase inhibitors (CST, #5872). One microliter of anti-FOXO1 antibody (CST, #2880) or anti-GFP antibody (Abcam, ab290) and 30 μl Dynabeads Protein G (Thermo Scientific, 10004D) were incubated overnight at 4 °C. Cell lysates were centrifuged and the protein concentration of the supernatants were quantitated using BCA protein assay kit (Thermo Scientific, 23227). The same amount of protein was incubated with antibody-Dynabeads conjugates for 4 h at 4 °C, then Dynabeads were washed in ice-cold PBS 3 times. Lamni buffer was added and the samples were denatured at 95 °C for 5 min. Aliquots of each protein lysate (1 mg) were subjected to SDS–polyacrylamide gel electrophoresis. Normal rabbit IgG (CST, #2729) was used as a negative control. After electrophoresis, proteins were transferred to nitrocellulose membranes and blocked for 30 min with 3% BSA in TBST (0.1% Tween 20 in TBS). Primary antibodies (diluted at a ratio of 1:1000 in blocking solution) were incubated overnight at 4 °C. After washes, membranes were incubated with anti-rabbit (CST, #7074) or anti-mouse (CST, #7076) secondary peroxidase-coupled antibody (diluted at a ratio of 1:5000 in TBST) for 1 h at RT. Target proteins were detected using ECL western blot detection solution (Millipore, WBKLS0500). Blot intensity was measured with ImageJ software. Primary antibodies used for immunoblotting were as follows: rabbit anti-phospho-MST1 (at Thr183) polyclonal (CST, #3681); mouse anti-MST1 monoclonal (BD biosciences, #611052); rabbit anti-MST1 monoclonal (CST, #14946); rabbit anti-phospho-LATS1 (at Thr1079) monoclonal (CST, #8654); rabbit anti-LATS1 monoclonal (CST, #3477); rabbit anti-phospho-YAP (at Ser127) polyclonal (CST, #4911); rabbit anti-YAP monoclonal (CST, #14074); rabbit anti-HIF1α monoclonal (CST, #14179); rabbit anti-phospho-AKT (at Ser473) monoclonal (CST, #4058); rabbit anti-AKT polyclonal (CST, #9272); rabbit anti-phospho-FOXO1 (at Ser256) polyclonal (CST, #9461); rabbit anti-phospho-VEGFR2 (at Tyr1175) monoclonal (CST, #2478); rabbit anti-VEGFR2 monoclonal (CST, #2479); rabbit anti-phospho-FOXO1 (at Ser212) polyclonal (Generated by Abclon); rabbit anti-FOXO1 monoclonal (CST, #2880); rabbit anti-β-actin monoclonal (Sigma-Aldrich, A5441); rabbit anti-GAPDH monoclonal (CST, #5174); rabbit anti-LAMIN B1 polyclonal (Abcam, ab16048); rabbit anti-GFP polyclonal (Abcam, ab290); mouse anti-FLAG monoclonal, horseradish peroxidase conjugated (Sigma-Aldrich, A8592). The uncropped and unprocessed scans with marker positions of all blots were included in the Source Data file.

### Fractionation of nuclear and cytoplasmic proteins

Cells were washed with ice-cold PBS and lysed with cytoplasmic extraction reagent (10 mM HEPES (pH 7.8), 10 mM KCL, 1.5 mM MgCl2, 0.5 mM 1,4-dithiothretiol, and protease inhibitor). Cells were scrape-loaded into pre-chilling tubes, incubated for 10 min on ice and spun at 15,000 *g* at 4 °C with 10% NP-40 solution. Then, supernatants were collected as the cytosolic fraction. Cell pellets were further washed with ice-cold PBS 2 times to remove cytosolic proteins. Washed cell pellets were suspended in lamni buffer as the nuclear fraction. Nuclear and cytosolic fraction were confirmed by immunoblotting for LAMIN B1 and GAPDH as nuclear and cytoplasmic markers, respectively.

### Isolation of mouse lung endothelial cells

After anesthesia, lungs were harvested at P6, cut into small pieces, and digested in buffer containing collagenase type 2 (Worthington), Dispase (Gibco, 17105041) and DNase I (Roche) at 37 °C for 30 min. Tissues were gently agitated, strained with a 100 μm nylon mesh to remove cell clumps, incubated in ACK lysis buffer for 2 min to remove erythrocytes and strained with a 40 μm nylon mesh. Single cell suspensions were incubated for 30 min with rat anti-CD31 APC-conjugated (BD Biosciences, 561814), rat anti-CD45 FITC-conjugated (eBioscience, #11-0451-81), and rat anti-TER119 FITC-conjugated (eBioscience, #11-5921-82). To discriminate dead cells, cells were stained with DAPI (Sigma-Aldrich) and cell sorting was performed with FACS AriaII (BD Biosciences). Data were analyzed using FlowJo software (Tree Star).

### RNA-sequencing analysis

HUVECs were cultured in 60 mm dish and transfected with GL2 (siCont-ECs), MST1 (siMST1-ECs) and FOXO1 (siFOXO1-ECs) siRNA. At 48 h after siRNA transfection, each siCont-, siMST1-, and siFOXO1-ECs were incubated under hypoxia for 3 h. After extracting total RNA from normoxic-ECs, hypoxic-ECs, siCont-ECs, siMST1-ECs, and siFOXO1-ECs using Trizol reagent (Invitrogen), 1 μg of total RNA was used to construct cDNA libraries with the TruSeq RNA library kit. The protocol consisted of poly A-selected RNA extraction, RNA fragmentation, random hexamer primed reverse transcription, and 100 nt paired-end sequencing by Illumina HiSeq 2500. The libraries were quantified using qPCR according to the qPCR Quantification Protocol Guide and qualified using an Agilent Technologies 2100 Bioanalyzer. The sequencing adapters were removed by Trimmomatic v0.26^61^. Then, the trimmed reads were mapped to the human Ensembl reference genome (GRCh38.91) using HISAT2 aligner^62^ so as to acquire the BAM file. Raw read counts were achieved from the following using the HT-Seq, then used to analyze the differentially expressed genes (DEG) between samples by applying EdgeR method. Hierarchically clustered heatmaps of DEG among siCont-ECs, siMST1-ECs, and siFOXO1-ECs were generated in R using the heatmap function. To analyze Gene Ontology biological process and get the Fold enrichment score and Q-score, the ingenuity pathway analysis (QIAGEN) was used and all DEGs were mapped to Gene Ontology database Released 2018-04-04 (http://www.geneontology.org/)^63,64^.

### RNA extraction and quantitative RT-PCR

Total RNA was extracted from samples using RNeasy mini kit (Qiagen) according the manufacturer’s protocols. A total of 1 μg of extracted RNA was transcribed into cDNA using GoScript^TM^ Reverse Transcription System (Promega). cDNA was mixed with primers and FastStart SYBR Green Master (Roche), and mRNA expression levels were measured by real-time PCR QuantStudio3 (Thermo Fisher Scientific). The primers were designed using Primer-BLAST. The list of qRT–PCR primers used in this study is described in Supplementary Table 1.

### Transcriptional profile analysis of microarray data

Gene set enrichment analysis (GSEA) was performed with v6.1 of the Molecular Signature Database (http://www.broadinstitute.org/gsea/msigdb), and the gene sets which were <0.05 nominal *P*-value were stated.

### OIR mouse model

Briefly, P7 mice were exposed to 75% oxygen in a hyperoxic chamber (COY laboratories, O2 Control InVivo Cabinet, Grass Lake, Michigan) for 5 days with their nursing mother and then returned to room air for 5 days.

### Code availability

R scripts used in the present work are available from the authors upon request.

### Statistics and reproducibility

No methods were not used to predetermine sample size. Reproducibility was ensured by performing more than five and three independent in vivo and in vitro experiments, respectively. Animals or samples were not randomized and the investigators were not blinded during experiments. Both male and female neonatal mice were analyzed at P6 and P12 and only male mice were used at 12 weeks of age. No animals were excluded from analysis. All values are presented as mean ± standard deviation (s.d.). Statistical significance was determined by the two-tailed unpaired *t*-test between 2 groups or the one-way ANOVA followed by Tukey’s honest significant difference (HSD) test with ranks for multiple-group comparison. Statistical analysis was performed using GraphPad Prism 7.0 (GraphPad Software). Statistical significance was set to *P*-value < 0.05.

### Reporting summary

Further information on experimental design is available in the Nature Research Reporting Summary linked to this article.

## Supplementary information

Supplementary Information

Description of Additional Supplementary Files

Supplementary Movie 1

Supplementary Movie 2

Supplementary Movie 3

Supplementary Movie 4

Supplementary Movie 5

Supplementary Movie 6

Reporting Summary

Source Data

## Data Availability

The RNA-sequencing data generated with this study have been deposited in Gene Expression Omnibus under the accession number GSE116033. The source data underlying all Figs and Supplementary Figs are provided as a Source Data file. A Reporting Summary for this article is available as a Supplementary Information file. All other data that support the findings of this study are available from the corresponding author upon reasonable request. Expression data from the published studies were obtained from the accession number GSE19284 in the Gene Expression Omnibus^39^.
