# Supplementary material for: A MST1–FOXO1 cascade establishes endothelial tip cell polarity and facilitates sprouting angiogenesis
Source: Nat Commun. 2019 Feb 19;10:838. doi: 10.1038/s41467-019-08773-2 (PMC6381131; doi:10.1038/s41467-019-08773-2)
Supplement: Supplementary file 1 — Supplementary Information [file 41467_2019_8773_MOESM1_ESM.pdf]

Supplementary Information

A MST1-FOXO1 cascade establishes endothelial tip cell polarity and facilitates sprouting angiogenesis

Yoo Hyung Kim, Jeongwoon Choi, Myung Jin Yang, Seon Pyo Hong, Choong-kun Lee, Yoshiaki Kubota, Dae-Sik Lim, Gou Young Koh

It includes;

1. Supplementary Figures 1-11 and their legends
2. Supplementary Table 1.

Supplementary Figure 1

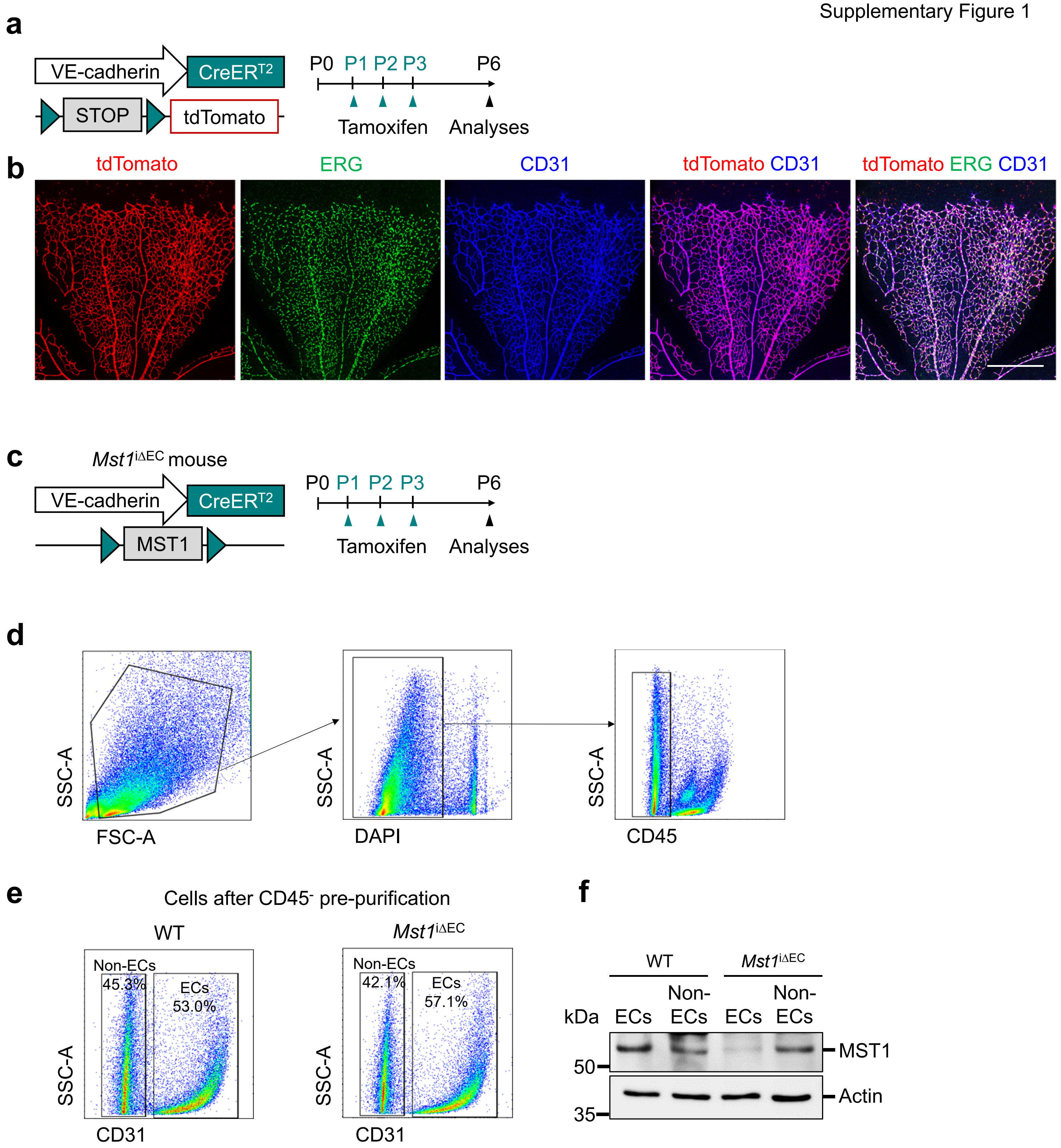

Supplementary Figure 1. Inducible EC-specific MST1 deletion in *Mst1*$^{i\Delta EC}$ mice.

(**a**) Diagram depicting the experimental schedule for monitoring EC-specific cre recombination using reporter mice with *VE-cadherin* Cre-ER^{T2} mice. (**b**) Images of tdTomato, ERG^{+} nuclei of EC and CD31^{+} retinal vessels. Scale bars, 500 µm. (**c**) Diagram depicting the experimental schedule for EC-specific deletion of MST1 in mouse lung ECs. (**d**) Gating strategy to sort CD45^{-} cells from WT and *Mst1*$^{i\Delta EC}$ mice. (**e**) Gating strategy to sort Non-ECs (CD45^{-}CD31^{-}) and ECs (CD45^{-}CD31^{+}) from WT and *Mst1*$^{i\Delta EC}$ mice. Numbers above and inside of bracketed lines indicate percentages of cells. (**f**) Immunoblot analyses of indicated proteins in sorted ECs and Non-ECs of WT and *Mst1*$^{i\Delta EC}$ mice. Source data are provided as a Source Data file.

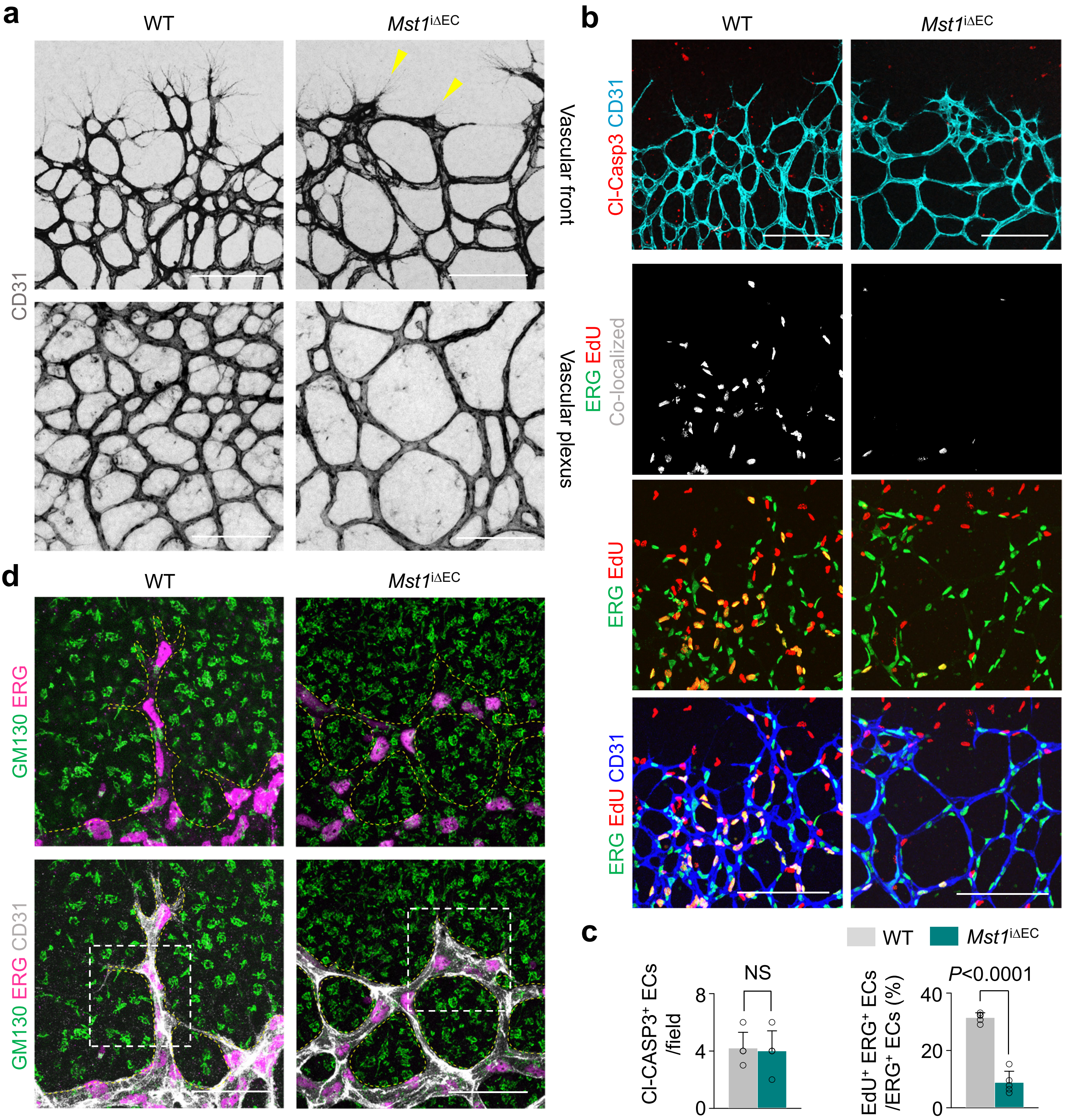

Supplementary Figure 2. Endothelial MST1 deletion impairs EC proliferation and polarization.
(**a**) Representative images of retinal vessels in the vascular front and plexus of WT and *Mst1*$^{i\Delta EC}$ mice. Note that *Mst1*$^{i\Delta EC}$ mice exhibit shortened sprouts (yellow arrowheads) with reduced vascular density and branching. Scale bars, 100 µm. (**b, c**) Images and comparisons of Cl-CASP3^{+} apoptotic ECs and EdU incorporated proliferating ECs in CD31^{+} vessels of WT (n=5) and *Mst1*$^{i\Delta EC}$ (n=5) mice. Scale bars, 100 µm. Data represent mean (bar) ± s.d. (error bars). *P* values, versus WT by two-tailed unpaired *t*-test. NS, not significant. (**d**) Images of CD31^{+} vessels, ERG^{+} nuclei of ECs and GM130^{+} Golgi apparatus at tip ECs of WT and *Mst1*$^{i\Delta EC}$ mice. The images of the insets (white dashed-line boxed) are magnified in Figure 1h. The yellow dashed line outlines CD31^{+} vessels. Scale bars, 50 µm. Source data are provided as a Source Data file.

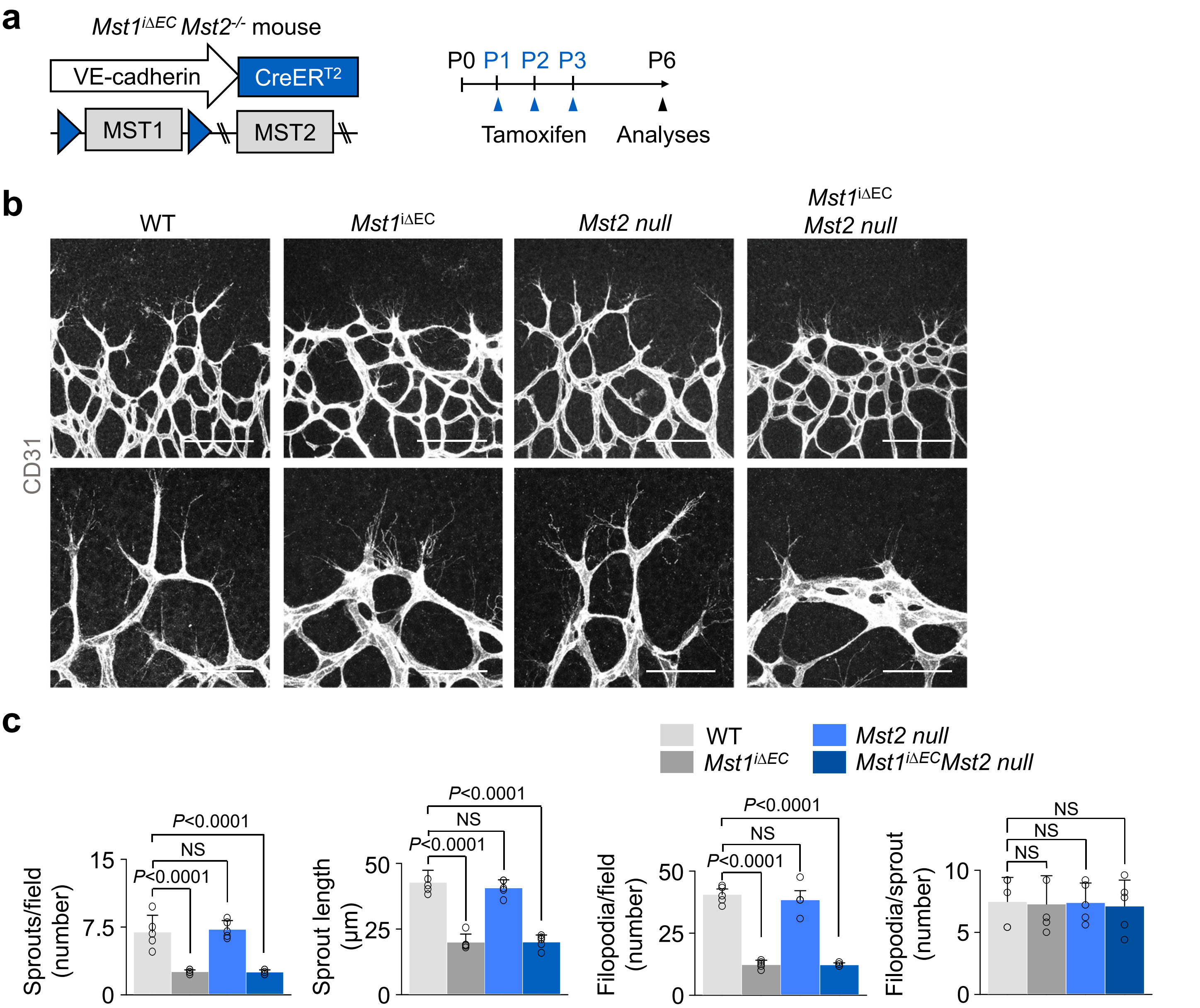

Supplementary Figure 3. Endothelial MST1 but not MST2 is critical in sprouting angiogenesis.
(**a**) Diagram depicting the experimental schedule for EC-specific deletion of MST1 in retinal vessels from P1 and/or constitutive knockout of MST2 and their analyses at P6. (**b, c**) Images of CD31$^+$ vessels and comparisons of indicated parameters. Scale bars, 100 µm (upper panels); 50 µm (lower panels). Data represent mean (bar) ± s.d. (error bars). *P* values, versus WT NS, not significant. Source data are provided as a Source Data file.

Supplementary Figure 4

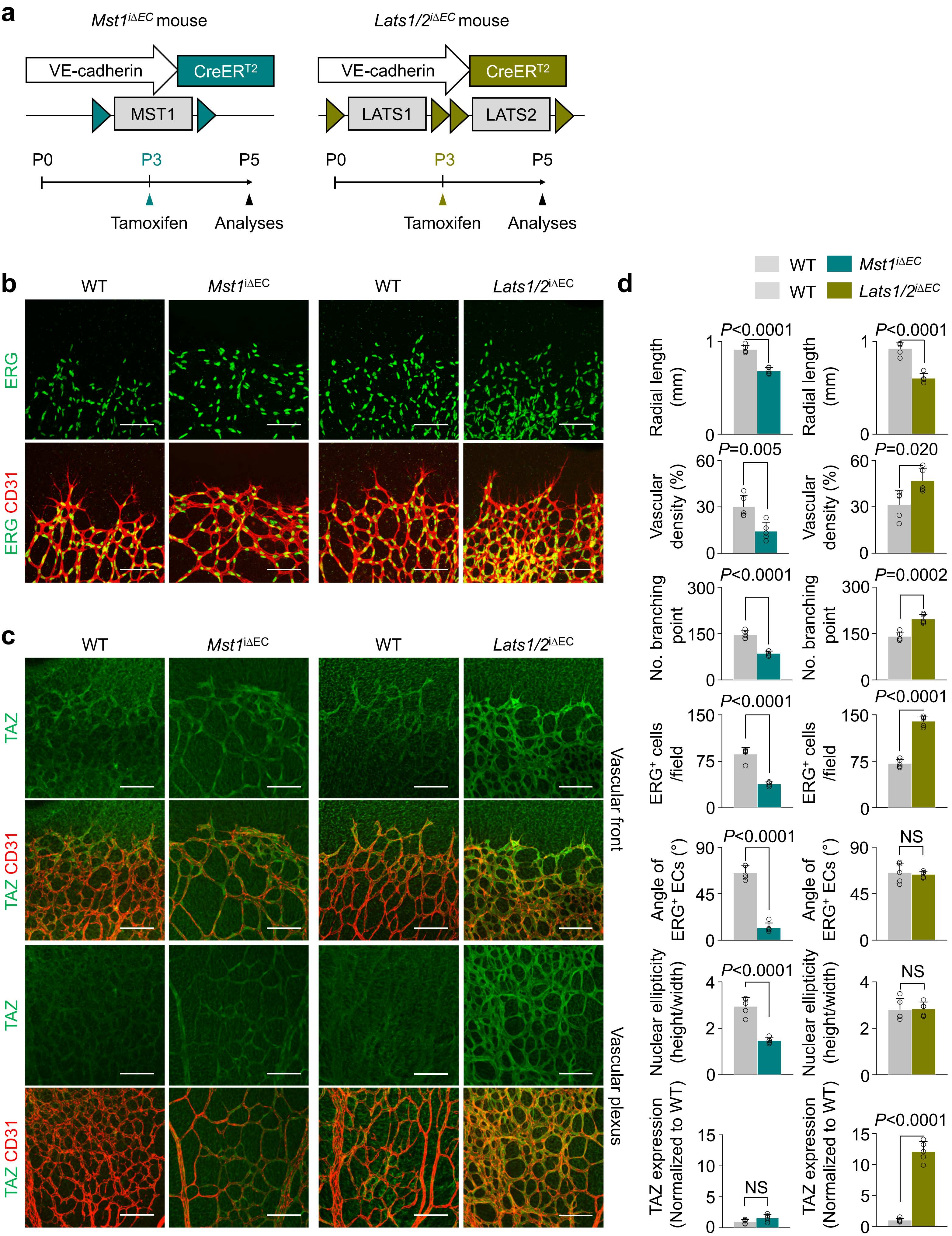

Supplementary Figure 4. Endothelial MST1 does not rely on the canonical Hippo pathway for sprouting angiogenesis.
(**a**) Diagram depicting the experimental schedule for EC-specific deletion of MST1 or LATS1/2 in retinal vessels from P3 and their analyses at P5. (**b**) Images of $CD31^+$ vessels and ERG^+ nuclei of ECs in WT, *Mst1*$^{i\Delta EC}$ and *Lats1/2*$^{i\Delta EC}$ mice. Scale bars, 100 µm. (**c**) Images of TAZ distribution in $CD31^+$ vessels at vascular front and plexus in WT, *Mst1*$^{i\Delta EC}$ and *Lats1/2*$^{i\Delta EC}$ mice. (**d**) Comparisons of indicated parameters in two of each WT (n=5), *Mst1*$^{i\Delta EC}$ (n=5), and *Lats1/2*$^{i\Delta EC}$ (n=5) mice. Data represent mean (bar) ± s.d. (error bars). *P* values, versus WT by two-tailed unpaired *t*-test. NS, not significant. Source data are provided as a Source Data file.

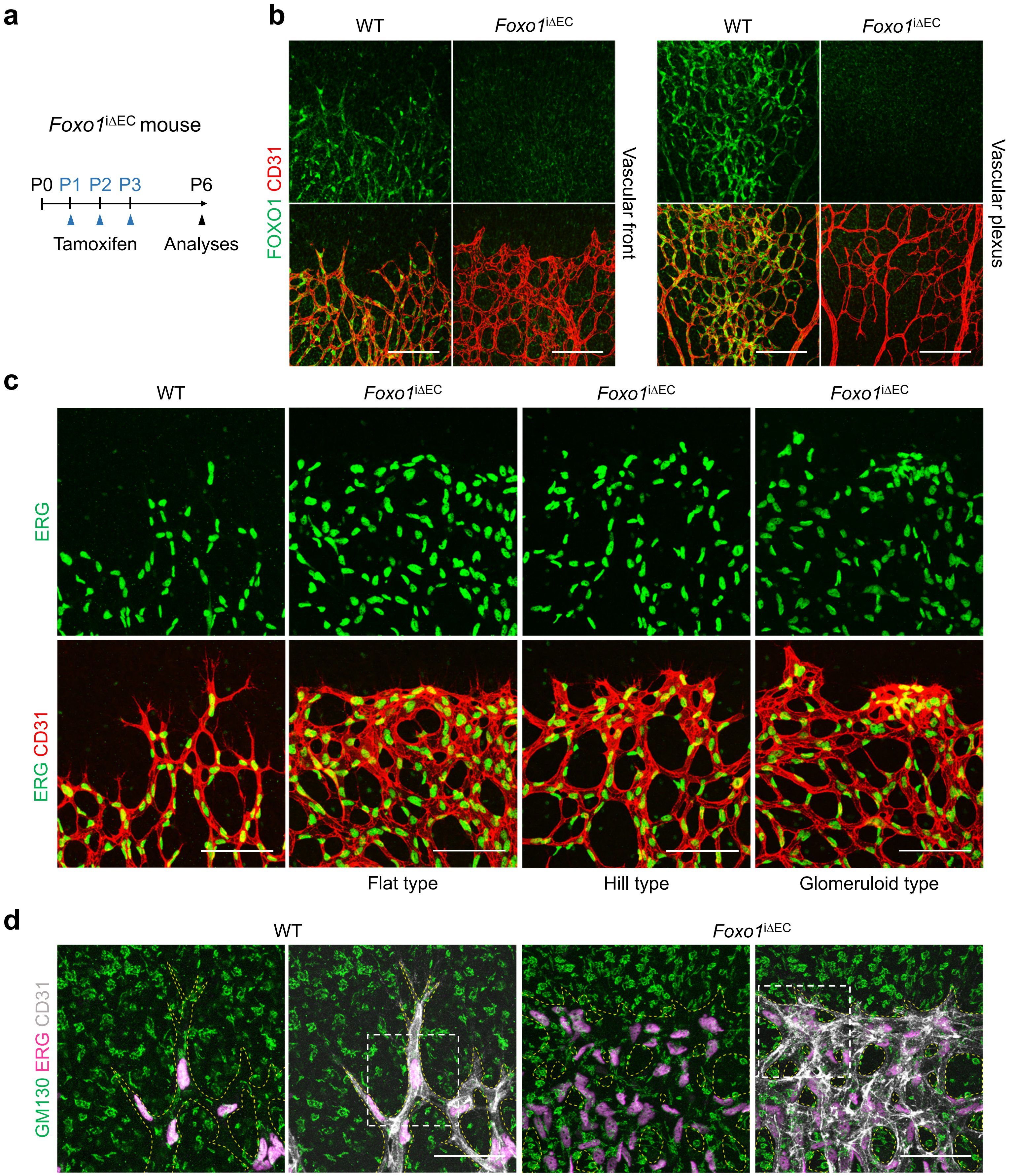

Supplementary Figure 5. Endothelial FOXO1 deletion impairs tip EC polarization.
(**a**) Diagram depicting the experimental schedule for EC-specific deletion of FOXO1 in retinal vessels from P1 and their analyses at P6. (**b**) Images of FOXO1 distribution in CD31^{+} vessels at P5 in WT and *Foxo1*$^{i\Delta EC}$ mice. Scale bars, 100 µm. (**C**) Images of CD31^{+} vessels and ERG^{+} nuclei of ECs showing variable sprouts morphology in *Foxo1*$^{i\Delta EC}$ mice. Scale bars, 50 µm. (**d**) Images of CD31^{+} vessels, ERG^{+} nuclei of ECs and GM130^{+} Golgi apparatus at tip ECs of WT and *Foxo1*$^{i\Delta EC}$ mice. The images of the inset (white dashed-line boxed) are magnified in Figure 4g. The yellow dashed line outlines CD31^{+} vessels. Scale bars, 50 µm.

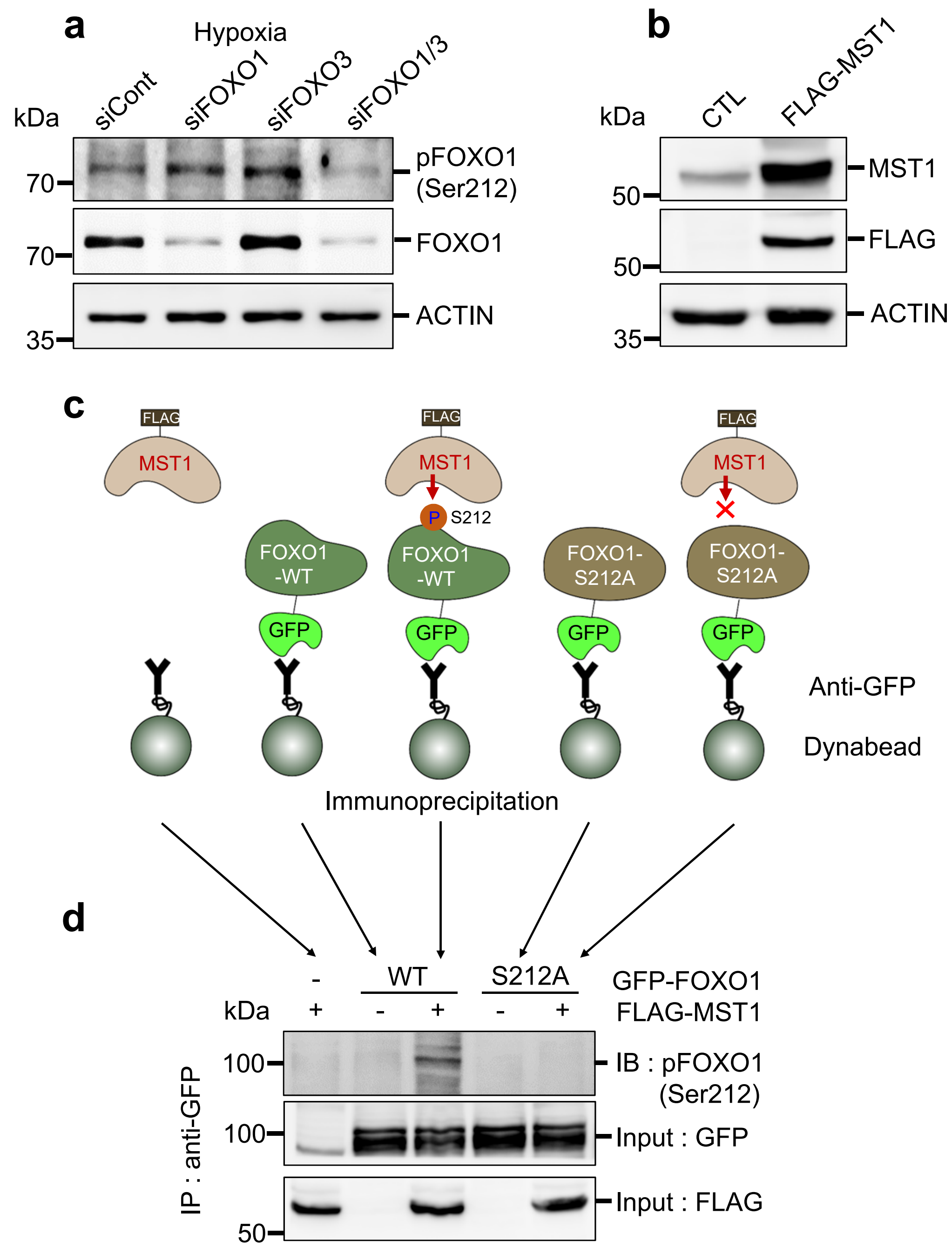

Supplementary Figure 6. The anti-phospho-FOXO1 polyclonal antibody recognizes FOXO1 phosphorylation at Serine 212.
(**a**) Immunoblot analysis of indicated proteins in siCont-ECs, siFOXO1-ECs, siFOXO3-ECs (HUVECs transfected with siRNA targeting *FOXO3* gene) and siFOXO1/3-ECs for the validation of anti-phospho-FOXO1 (Ser212) antibody. (**b**) Immunoblot analysis of indicated proteins in HEK293T cells transfected with either control vector (CTL) or gene construct encoding MST1 (FLAG-MST1). (**c**) Schematic picture depicting our approach to validate anti-phospho-FOXO1 (Ser212) antibody. (**d**) Immunoprecipitation analysis in HEK293T cells with anti-GFP antibody followed by immunoblotting with anti-phospho-FOXO1 (Ser212) antibody. HEK293T cells were transfected with gene constructs encoding either GFP-tagged FOXO1 (GFP-FOXO1-WT) or non-phosphorylatable FOXO1 (GFP-FOXO1-S212A) together with either CTL or FLAG-MST1 prior to immunoprecipitation. Source data are provided as a Source Data file.

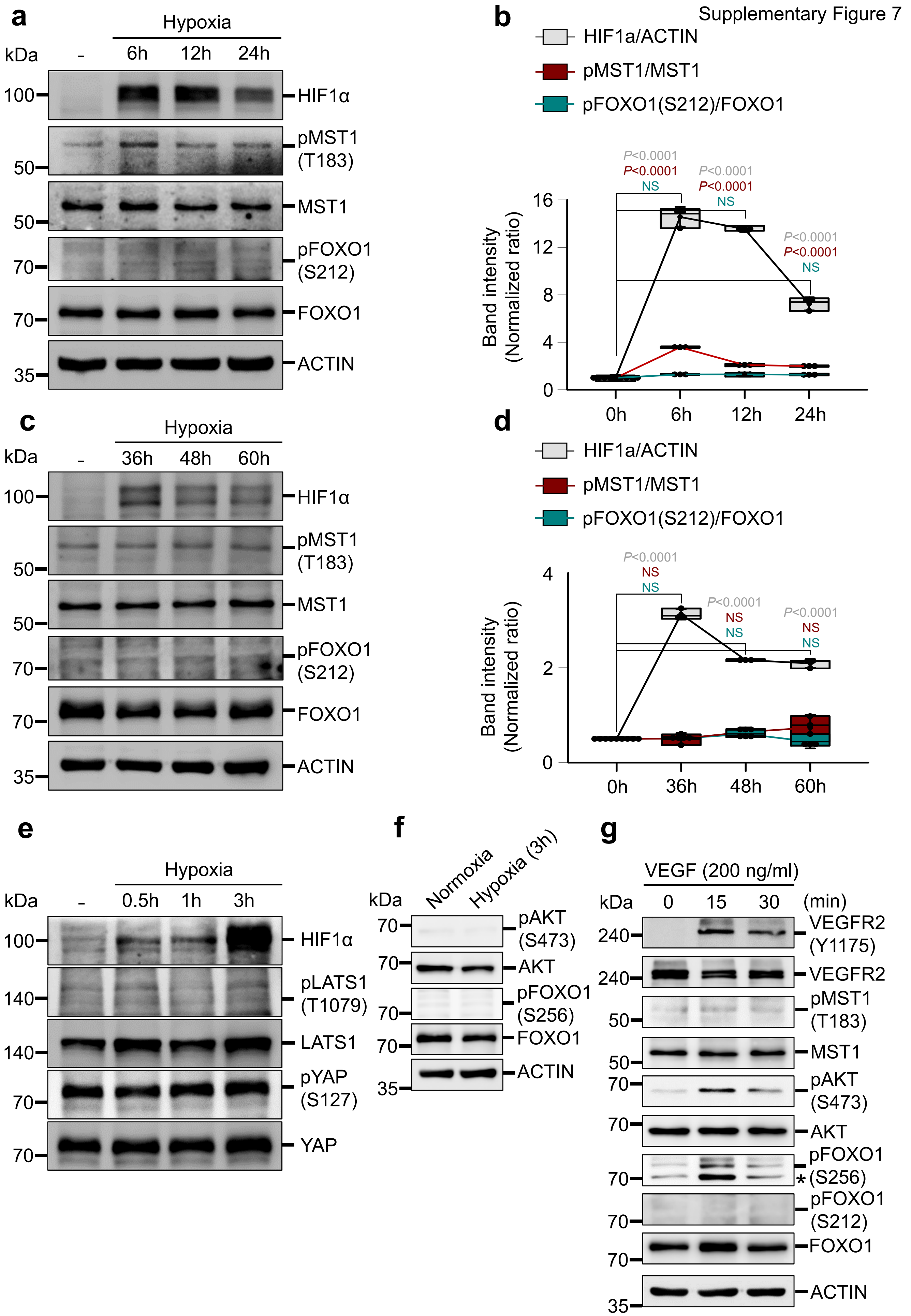
a
Hypoxia
kDa
-
6h
12h
24h
100
HIF1α
pMST1
(T183)
50
MST1
50
pFOXO1
(S212)
70
FOXO1
70
ACTIN
35
b
HIF1a/ACTIN
pMST1/MST1
pFOXO1(S212)/FOXO1
Band intensity
(Normalized ratio)
P<0.0001
P<0.0001
NS
0h
6h
12h
24h
c
Hypoxia
36h
48h
60h
d
0h
36h
48h
60h
e
Hypoxia
0.5h
1h
3h
pLATS1
(T1079)
LATS1
pYAP
(S127)
YAP
140
f
Normoxia
Hypoxia (3h)
pAKT
(S473)
AKT
pFOXO1
(S256)
g
VEGF (200 ng/ml)
0
15
30
(min)
VEGFR2
(Y1175)
VEGFR2
240
*

Supplementary Figure 7. Hypoxia activates the MST1-FOXO1 cascade in primary cultured HUVECs
(**a-d**) Immunoblot analyses and comparisons of indicated proteins in HUVECs exposed to hypoxia (1% O_2) for indicated times (n = 3, each group). Center line, median; Box limits, upper and lower quartiles; Whiskers, s.d. *P* values versus 0 h by one-way ANOVA with Tukey`s *post hoc* test. NS, not significant. (**e**-**g**) Immunoblot analyses of indicated proteins in HUVECs exposed to hypoxia (1% O_2) or VEGF (200 ng/ml) for indicated times. *, nonspecific band.

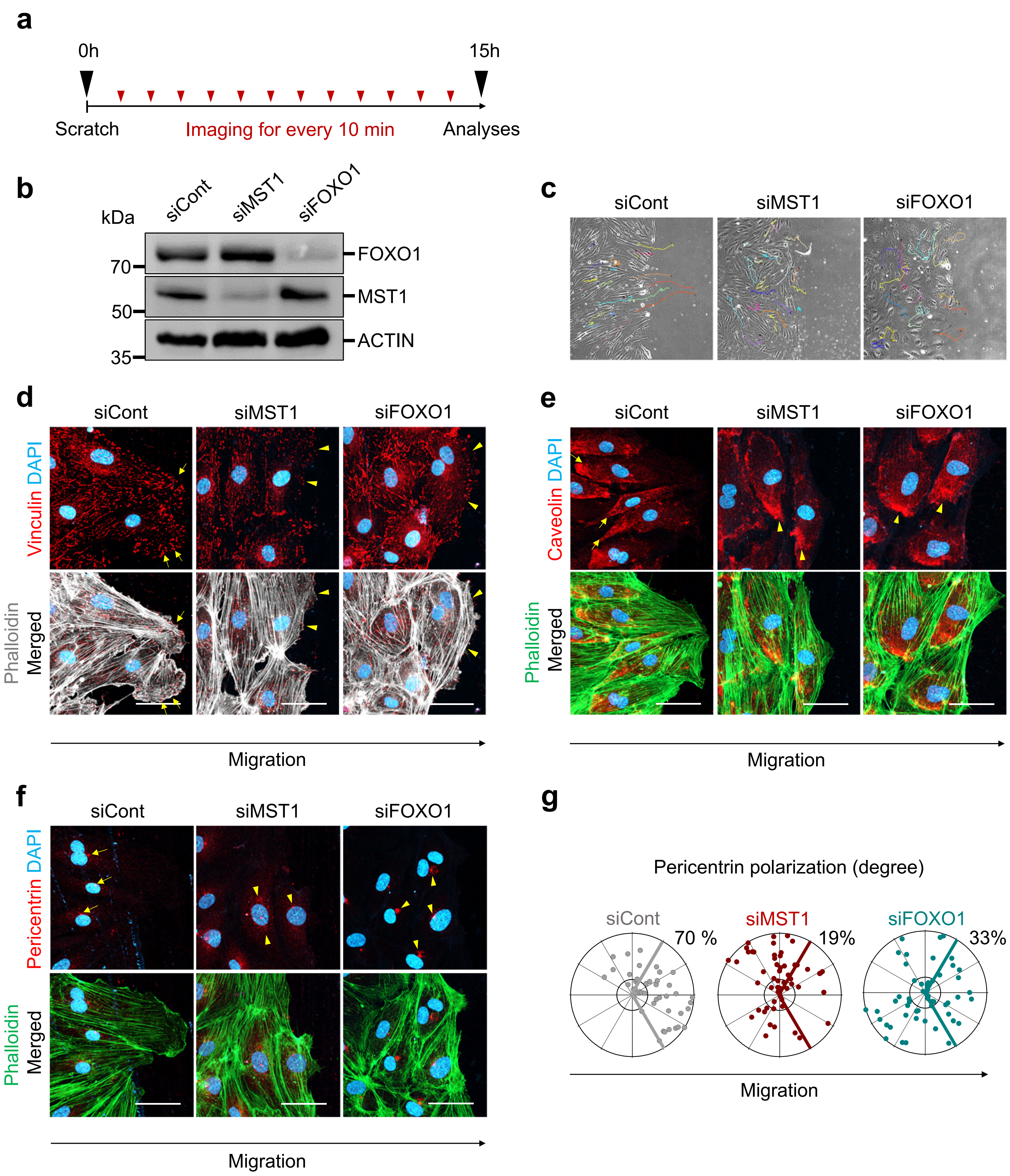
a
0h
15h
Scratch
Imaging for every 10 min
Analyses
b
kDa
siCont
siMST1
siFOXO1
FOXO1
MST1
ACTIN
70
50
35
c
siCont
siMST1
siFOXO1
d
siCont
siMST1
siFOXO1
Vinculin DAPI
Phalloidin Merged
Migration
e
siCont
siMST1
siFOXO1
Caveolin DAPI
Phalloidin Merged
Migration
f
siCont
siMST1
siFOXO1
Pericentrin DAPI
Phalloidin Merged
Migration
g
Pericentrin polarization (degree)
siCont
70 %
siMST1
19%
siFOXO1
33%
Migration

Supplementary Figure 8. MST1-FOXO1 cascade regulates cell polarity during directional EC migration
(**a**) Diagram depicting the experimental schedule for a wound scratch assay with time lapse imaging every 10 min for 15 h. (**b**) Immunoblot analyses in siCont-ECs, siMST1-ECs and siFOXO1-ECs to confirm depletion of the corresponding proteins. (**c**) Images of cell tracked line every 30 min in indicated ECs. n=19(siCont), 20(siMST1), 17(siFOXO1). Similar findings were observed in three independent experiments. (**d**) Representative images showing phalloidin^{+} actin cytoskeleton, vinculin and DAPI in the leading edge of indicated ECs at 9 h after initiating cell migration. Note that vinculin incorporated focal adhesion (yellow arrowheads) and lamellipodia (yellow arrowheads) are rarely developed in siMST1-ECs and siFOXO1-ECs compared to siCont-ECs (yellow arrows). Scale bars, 50 µm. (**e**) Representative images showing phalloidin^{+} actin cytoskeleton, caveolin and DAPI in the leading edge of indicated ECs at 9 h after initiating cell migration. Note that caveolin (yellow arrows) in siCont-ECs are localized in the opposite direction of cell migration, while caveolin (yellow arrowheads) in siMST1-ECs and siFOXO1-ECs are localized randomly. Scale bars, 50 µm. (**f**) Representative images showing phalloidin^{+} actin cytoskeleton, pericentrin^{+} centrosome and DAPI in the leading edge of indicated ECs at 9 h after initiating cell migration. Note that pericentrin^{+} centrosome (yellow arrows) in siCont-ECs are localized in the direction of cell migration, while pericentrin^{+} centrosome (yellow arrowheads) in siMST1-ECs and siFOXO1-ECs are localized randomly. Scale bars, 50 µm. (**g**) Polar plots showing pericentrin polarization. n=45(siCont), 57(siMST1), 54(siFOXO1). The bold lines indicate 120° region centered on the vector which is vertical to the scratch direction. The numbers indicate the frequency of dots within the 120° region of the bold line. Source data are provided as a Source Data file.

Supplementary Figure 9

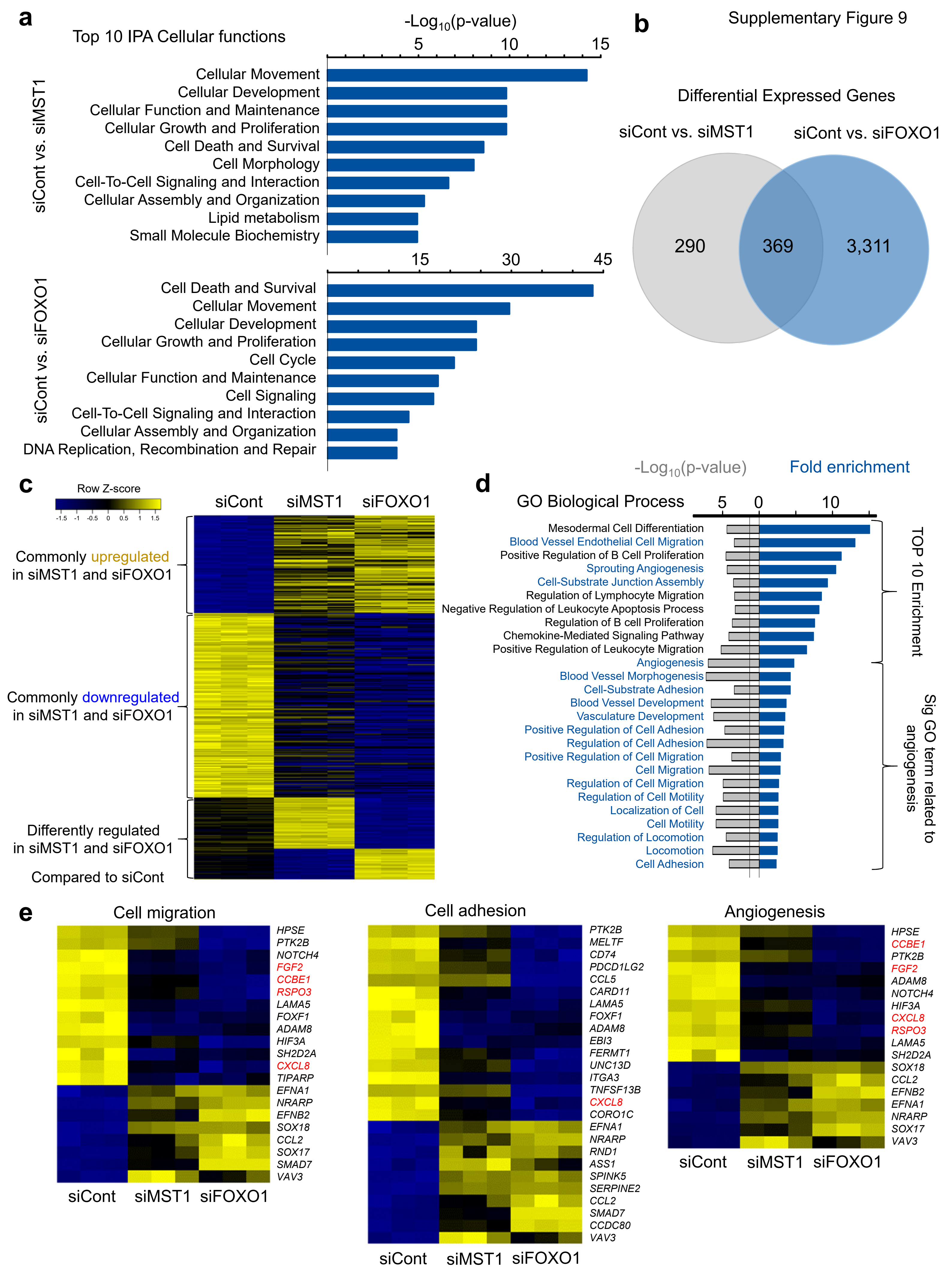

Supplementary Figure 9. MST1-FOXO1 cascade regulates gene expression related to cell polarity.
(**a**) Gene Ontology (GO) term analysis using IPA on the RNA sequencing data in comparison of siCont-ECs versus siMST1-ECs or siFOXO1-ECs (**b**) Venn-diagram depicting commonly regulated genes identified by differentially expressed gene analysis in siCont-ECs versus siMST1-ECs or siFOXO1-ECs. Numbers inside the Venn-diagram indicates the number of genes. (**c**) Clustered heat map of commonly regulated genes by MST1 and FOXO1. 286 of 369 genes are commonly up- or down regulated by MST1 and FOXO1. (**d**) GO term enrichment analysis for the commonly up- or down regulated genes by MST1 and FOXO1. False discovery rate ≤ 0.05. (**e**) Representative clustered heat map of 'Cell migration', 'Cell adhesion' and 'Angiogenesis'. Genes marked in red are those with high fold change compared with siCont-ECs.

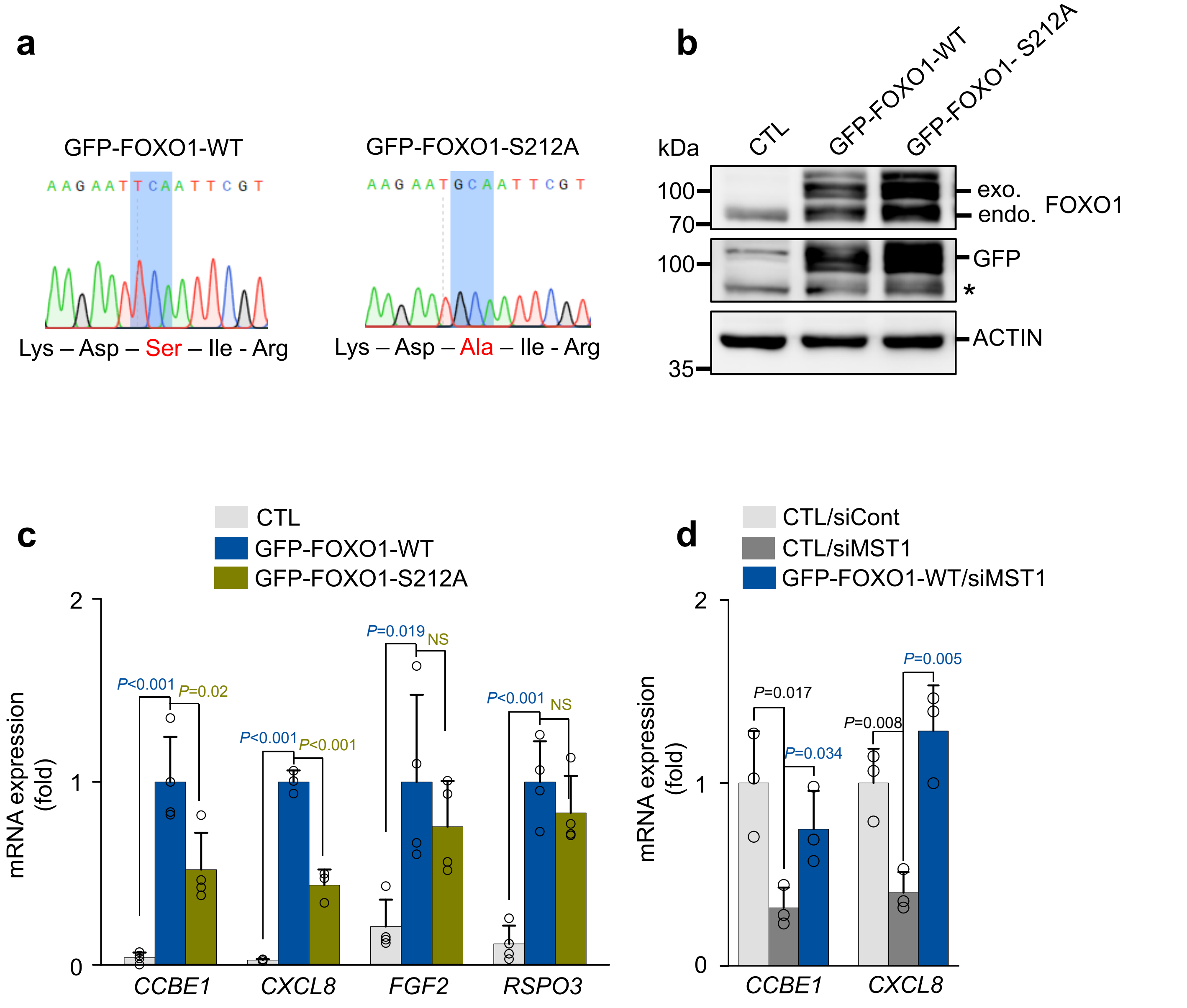

Supplementary Figure 10. MST1-FOXO1 cascade transcriptionally regulates the expressions of *CCBE1* and *CXCL8*. (**a**) Diagram depicting DNA and protein sequences of gene constructs encoding FOXO1 (GFP-FOXO1-WT) and non-phosphorylatable FOXO1 (GFP-FOXO1-S212A). (**b**) Immunoblot analysis of indicated proteins in HUVECs transfected with gene constructs of control (CTL), GFP-FOXO1-WT, or GFP-FOXO1-S212A. *, nonspecific band. (**c**) Quantitative PCR analyses of indicated gene expressions in HUVECs transfected with gene constructs of CTL, GFP-FOXO1-WT, or GFP-FOXO1-S212A. Data represent mean (bar) ± s.d. (error bars). *P* values (blue), CTL versus GFP-FOXO1-WT by one-way ANOVA with Tukey`s *post hoc* test. *P* values (green), GFP-FOXO1-WT versus GFP-FOXO1-S212A by one-way ANOVA with Tukey`s *post hoc* test. (**d**) Quantitative PCR analyses of *CCBE1* and *CXCL8* gene expression in siCont-ECs transfected with gene constructs of CTL or siMST1-ECs transfected with gene constructs of CTL or GFP-FOXO1-WT. Data represent mean (bar) ± s.d. (error bars). *P* values (black), CTL/siCont versus CTL/siMST1 by one-way ANOVA with Tukey`s *post hoc* test. *P* values (blue), CTL/siMST1 versus GFP-FOXO1-WT/siMST1 by one-way ANOVA with Tukey`s *post hoc* test. NS, not significant. Source data are provided as a Source Data file.

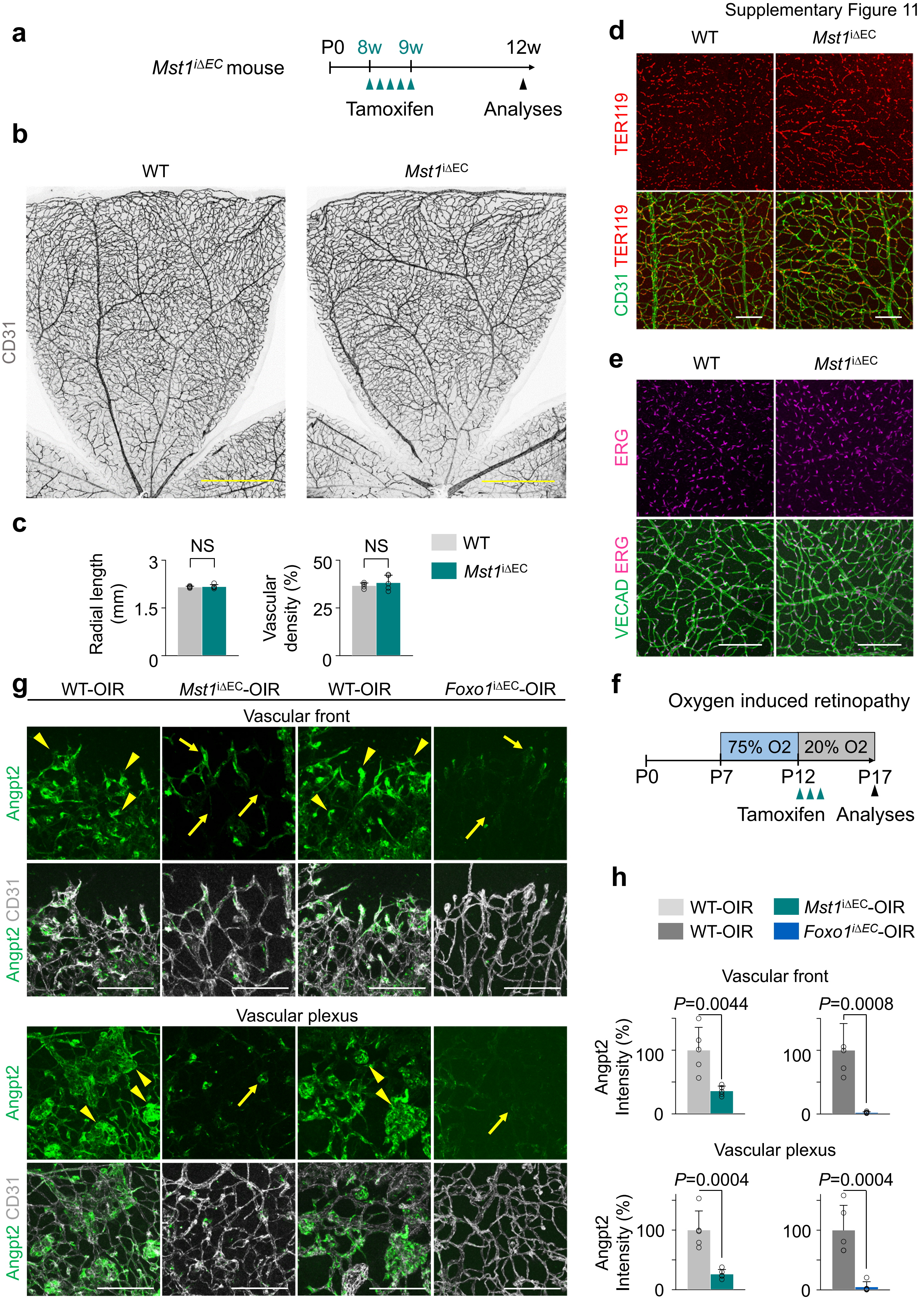
a
Mst1iΔEC mouse
P0 8w 9w 12w
Tamoxifen
Analyses
b
WT
Mst1iΔEC
CD31
c
Radial length (mm)
Vascular density (%)
NS
WT
Mst1iΔEC
d
WT
Mst1iΔEC
TER119
CD31 TER119
e
ERG
VECAD ERG
f
Oxygen induced retinopathy
75% O2
20% O2
P0 P7 P12 P17
Tamoxifen
Analyses
g
WT-OIR
Mst1iΔEC-OIR
Foxo1iΔEC-OIR
Vascular front
Vascular plexus
Angpt2
Angpt2 CD31
h
WT-OIR
Mst1iΔEC-OIR
Foxo1iΔEC-OIR
Angpt2 Intensity (%)
P=0.0044
P=0.0008
P=0.0004
P=0.0004

Supplementary Figure 11. Endothelial MST1 is dispensable for vessel maintenance, but MST1-FOXO1 cascade is required for pathological angiogenesis.
(**a**) Diagram depicting the experimental schedule for EC-specific deletion of MST1 in retinal vessels of 8-week-old mice and their analysis after 4 weeks in *Mst1*$^{i\Delta EC}$ mice. (**b, c**) Images of CD31^{+} retinal vessels and comparisons of indicated parameters in WT (n=5) and *Mst1*$^{i\Delta EC}$ (n=5) mice. Scale bars, 500 µm. (**d**) Images of TER119^{+} RBC and CD31^{+} vessels. No visible hemorrhage is detected in both mice. Scale bars, 100 µm. (**e**) Images of VECAD and ERG^{+} nuclei of ECs. No abnormal alignment of ECs nuclei is observed in both mice. Scale bars, 100 µm. (**f**) Diagram depicting the experimental schedule for generation of oxygen-induced retinopathy model (OIR) in WT, *Mst1*$^{i\Delta EC}$, and *Foxo1*$^{i\Delta EC}$ mice. (**g, h**) Images of CD31^{+} vessels and Angpt2 expression and comparisons of Angpt2 intensity in tip ECs and NVT ECs in WT-OIR (n=5), *Mst1*$^{i\Delta EC}$-OIR (n=5), and *Foxo1*$^{i\Delta EC}$-OIR (n=5) mice. Note that Angpt2 expression is reduced in tip ECs and NVT ECs in *Mst1*$^{i\Delta EC}$-OIR and *Foxo1*$^{i\Delta EC}$-OIR (yellow arrows) compared with that in WT-OIR mice (yellow arrowheads). Scale bars, 100 µm. Data represent mean (bar) ± s.d. (error bars). *P* values, versus WT by two-tailed unpaired *t*-test. Source data are provided as a Source Data file.

Supplementary Table 1. List of Primer Sets for Quantitative Real-Time RT-PCR for human samples

Name	Sequence (5' - 3')	
CCBE1	Forward	CACATTAAGCAAGGCCGGAG
	Reverse	TCCTCTCTCCCCCTTAGAACC
CXCL8	Forward	CATACTCCAAACCTTTCCACC
	Reverse	AGCTTTACAATAATTTCTGTGTTGG
FGF2	Forward	TGGTATGTGGCACTGAAACG
	Reverse	TATAGCTTTCTGCCCAGGTCC
RSPO3	Forward	ACAATTGCCCAGAAGGGTTG
	Reverse	AGTCCCTCTTTTGAAGCCAC
GAPDH	Forward	CCACTCCTCCACCTTTGACG
	Reverse	TTCGTTGTCATACCAGGAAATGAG
