# Supplementary material for: A MST1–FOXO1 cascade establishes endothelial tip cell polarity and facilitates sprouting angiogenesis
Source: Nat Commun. 2019 Feb 19;10:838. doi: 10.1038/s41467-019-08773-2 (PMC6381131; doi:10.1038/s41467-019-08773-2)
Supplement: Supplementary file 2 — Description of Additional Supplementary Files [file 41467_2019_8773_MOESM2_ESM.docx]

**Description of Supplementary Files**

**File Name:** Supplementary Movie 1.

**Description:** Time lapse imaging of cell migration in siCont‐ ECs.  A representative video of a wound scratch assay with time lapse imaging every 10 min for 15 h using HUVECs transfected with siRNA for control. Similar findings were observed in 3 independent experiments.

**File Name:** Supplementary Movie 2.

**Description:** Time lapse imaging of cell migration in siMST1‐ECs.  A representative video of a wound scratch assay with time lapse imaging every 10 min for 15 h using HUVECs transfected with siRNA for MST1 gene. Similar findings  were observed in 3 independent experiments.

**File Name:** Supplementary Movie 3.

**Description:** Time lapse imaging of cell migration in siFOXO1‐ECs.  A representative video of a wound scratch assay with time lapse imaging every 10 min for 15 h using HUVECs transfected with siRNA for FOXO1 gene. Similar findings were observed in 3 independent experiments.

**File Name:** Supplementary Movie 4.

**Description:** Time lapse imaging of cell migration tracking in siCont‐ECs. A representative video of a wound scratch assay with time lapse imaging every 30 min for 15 h using HUVECs transfected with siRNA for control. Similar findings were observed in 3 independent experiments.

**File Name:** Supplementary Movie 5.

**Description:** Time lapse imaging of cell migration tracking in siMST1‐ECs. A representative video of a wound scratch assay with time lapse imaging every 30 min for 15 h using HUVECs transfected with siRNA for MST1 gene. Similar findings were observed in 3 independent experiments.

**File Name:** Supplementary Movie 6.

**Description:** Time lapse imaging of cell migration tracking in siFOXO1‐ECs. A representative video of a wound scratch assay with time lapse imaging every 30 min for 15 h using HUVECs transfected with siRNA for FOXO1 gene. Similar findings were observed in 3 independent experiments.
