# Supplementary material for: A MST1–FOXO1 cascade establishes endothelial tip cell polarity and facilitates sprouting angiogenesis
Source: Nat Commun. 2019 Feb 19;10:838. doi: 10.1038/s41467-019-08773-2 (PMC6381131; doi:10.1038/s41467-019-08773-2)
Supplement: Supplementary file 9 — Reporting Summary [file 41467_2019_8773_MOESM9_ESM.pdf]

nature research

Corresponding author(s): Gou Young Koh

Last updated by author(s): Dec 26, 2018

Reporting Summary

Nature Research wishes to improve the reproducibility of the work that we publish. This form provides structure for consistency and transparency in reporting. For further information on Nature Research policies, see Authors & Referees and the Editorial Policy Checklist.

Statistics

For all statistical analyses, confirm that the following items are present in the figure legend, table legend, main text, or Methods section.

n/a	Confirmed	
☐	☒	The exact sample size (*n*) for each experimental group/condition, given as a discrete number and unit of measurement
☐	☒	A statement on whether measurements were taken from distinct samples or whether the same sample was measured repeatedly
☐	☒	The statistical test(s) used AND whether they are one- or two-sided<br>*Only common tests should be described solely by name; describe more complex techniques in the Methods section.*
☐	☒	A description of all covariates tested
☐	☒	A description of any assumptions or corrections, such as tests of normality and adjustment for multiple comparisons
☐	☒	A full description of the statistical parameters including central tendency (e.g. means) or other basic estimates (e.g. regression coefficient) AND variation (e.g. standard deviation) or associated estimates of uncertainty (e.g. confidence intervals)
☐	☒	For null hypothesis testing, the test statistic (e.g. *F*, *t*, *r*) with confidence intervals, effect sizes, degrees of freedom and *P* value noted<br>*Give P values as exact values whenever suitable.*
☒	☐	For Bayesian analysis, information on the choice of priors and Markov chain Monte Carlo settings
☒	☐	For hierarchical and complex designs, identification of the appropriate level for tests and full reporting of outcomes
☒	☐	Estimates of effect sizes (e.g. Cohen's *d*, Pearson's *r*), indicating how they were calculated

Our web collection on statistics for biologists contains articles on many of the points above.

Software and code

Policy information about availability of computer code

Data collection	Image data was collected from LSM image software (Zeiss) and Zen software (Zeiss). FACS DIVA (v8.0.1) was used for sorts. QuantStudio Design & Analysis Software v1.4 was used for RT-PCR.
Data analysis	See "Methods" for detailed analysis protocol. For image analyses, ZEN (black 2.3 SP1), Imaris x64 (8.2.1), ImageJ (1.50i), and Adobe photoshop (CC 2017) were used. For sorting data analysis, FlowJo (V10) was used. For RNA sequencing data analyses, GSEA v3.0, Ingenuity pathway analysis (QIAGEN, version 01-07), Trimmomatic v0.26, HISAT2 aligner, EdgeR, and Gene Ontology database released 2018-04-04 were used. For cell migration analyses, CellTracker v1.1 implemented in MATLAB R2017a was used. For statistical analyses, Sigmaplot v12.5 and GraphPad Prism 7 were used.

For manuscripts utilizing custom algorithms or software that are central to the research but not yet described in published literature, software must be made available to editors/reviewers. We strongly encourage code deposition in a community repository (e.g. GitHub). See the Nature Research guidelines for submitting code & software for further information.

Data

Policy information about availability of data

All manuscripts must include a data availability statement. This statement should provide the following information, where applicable:

- Accession codes, unique identifiers, or web links for publicly available datasets
- A list of figures that have associated raw data
- A description of any restrictions on data availability

The RNA-sequencing data generated with this study have been deposited in Gene Expression Omnibus under the accession number GSE116033 [https://www.ncbi.nlm.nih.gov/geo/query/acc.cgi?acc=GSE116033]. The source data underlying all Figs and Supplementary Figs are provided as a Source Data file. All other data that support the findings of this study are available from the corresponding author upon reasonable request.

Field-specific reporting

Please select the one below that is the best fit for your research. If you are not sure, read the appropriate sections before making your selection.

☒ Life sciences ☐ Behavioural & social sciences ☐ Ecological, evolutionary & environmental sciences

For a reference copy of the document with all sections, see nature.com/documents/nr-reporting-summary-flat.pdf

Life sciences study design

All studies must disclose on these points even when the disclosure is negative.

Sample size	No statistical method was used to predetermine sample size. Sample size were determined based on previous studies in the field to enable statistical analyses and ensure reproducibility.
Data exclusions	No data were excluded from this study.
Replication	For in vivo analyses, more than five independent experiments were replicated. For in vitro analyses, more than three independent experiments were replicated.
Randomization	Animals or samples were not randomized. Both male and female neonatal mice were analysed at P6 and P12 and only male mice were analysed at 12-week.
Blinding	The investigators were not blinded during experiments. However, two independent investigators have performed most of in vivo analyses in parallel. For RNA sequencing, independent investigator analysed data in unsupervised manner.

Reporting for specific materials, systems and methods

We require information from authors about some types of materials, experimental systems and methods used in many studies. Here, indicate whether each material, system or method listed is relevant to your study. If you are not sure if a list item applies to your research, read the appropriate section before selecting a response.

Materials & experimental systems

n/a	Involved in the study	
☐	☒	Antibodies
☐	☒	Eukaryotic cell lines
☒	☐	Palaeontology
☐	☒	Animals and other organisms
☒	☐	Human research participants
☒	☐	Clinical data

Methods

n/a	Involved in the study	
☒	☐	ChIP-seq
☐	☒	Flow cytometry
☒	☐	MRI-based neuroimaging

Antibodies

Antibodies used

For immunofluorescence: hamster anti-CD31 monoclonal (Millipore, MAB1398Z); isolectin B4 (IB4), Alexa Fluor 594-conjugated (Thermo Fisher Scientific, I21413); rat anti-VE-cadherin monoclonal (BD Pharmingen, 555289); mouse anti-VE-cadherin monoclonal (BD Biosciences, #555661); rabbit anti-ERG monoclonal (Abcam, ab92513); rabbit anti-cleaved-caspase-3 (Cl-CASP3) polyclonal (CST, #9664); rabbit anti-pHH3 polyclonal (Millipore, 05-806);
rabbit anti-collagen IV polyclonal (Bio-Rad, 2150-1470); rat anti-ICAM2 monoclonal (BD Pharmingen, 553326); rat anti-TER119 monoclonal (BD Pharmingen, 561033); rabbit anti-GLUT1 polyclonal (EMD Millipore, 07-1401); mouse anti-GM130 monoclonal, Alexa Fluor 647-conjugated (BD Biosciences , #558712); rabbit anti-TAZ polyclonal (Sigma-Aldrich, HPA007415); rabbit anti-FOXO1 monoclonal (CST, #2880); human anti-Angpt2 monoclonal (clone 4H10)58; Phalloidin, Alexa Fluor 488- conjugated (Thermo Fisher Scientific, A12379); Phalloidin, Alexa Fluor 594-conjugated (Thermo Fisher Scientific, A12381); mouse anti-caveolin-1 monoclonal (Abcam, ab17052); rabbit anti-GM130 polyclonal, Alexa Fluor 555-conjugated (Thermo Fisher Scientific, PA1-077-A555); rabbit anti-GM130 polyclonal, Alexa Fluor 647-conjucated (Thermo Fisher Scientific, PA1-077-A647); mouse anti-alpha tubulin monoclonal, Alexa Fluor 488-conjugated (Thermo Fisher Scientific, #322588); mouse anti-vinculin monoclonal, Alexa Fluor 488-conjugated (Thermo Fisher Scientific, #53-9777-80); mouse anti-pericentrin monoclonal (Abcam, ab28144).

For immunoblot: rabbit anti-phospho-MST1 (at Thr183) polyclonal (CST, #3681); mouse anti-MST1 monoclonal (BD biosciences, #611052); rabbit anti-MST1 monoclonal (CST, #14946); rabbit anti-phospho-LATS1 (at Thr1079) monoclonal (CST, #8654); rabbit anti-LATS1 monoclonal (CST, #3477); rabbit anti-phospho-YAP (at Ser127) polyclonal (CST, #4911); rabbit anti-YAP monoclonal (CST, #14074); rabbit anti-HIF1α monoclonal (CST, #14179); rabbit anti-phospho-AKT (at Ser473) monoclonal (CST, #4058); rabbit anti-AKT polyclonal (CST, #9272); rabbit anti-phospho-FOXO1 (at Ser256) polyclonal (CST, #9461); rabbit anti-phospho-VEGFR2 (at Tyr1175) monoclonal (CST, #2478); rabbit anti-VEGFR2 monoclonal (CST, #2479); rabbit anti-phospho-FOXO1 (at Ser212) polyclonal (Generated by Abclon); rabbit anti-FOXO1 monoclonal (CST, #2880); rabbit anti-β-actin monoclonal (Sigma-Aldrich,

	A5441); rabbit anti-GAPDH monoclonal (CST, #5174); rabbit anti-LAMIN B1 polyclonal (Abcam, ab16048); rabbit anti-GFP polyclonal (Abcam, ab290); mouse anti-FLAG monoclonal, horseradish peroxidase conjugated (Sigma-Aldrich, A8592).
Validation	These antibodies have either validated by manufacturer or previous published literatures in our group. The anti-phospho-FOXO1 at Ser212 polyclonal antibody validated in published literature (Lehtinen MK, et al. Cell. 2006; Yuan Z et al. J Biol Chem. 2009) and by our group (presented in Supplementary Figure 6).

Eukaryotic cell lines

Policy information about cell lines

Cell line source(s)	Pooled primary cultured HUVEC cells were purchased from Lonza (C2519A).
Authentication	HUVEC were authentificated based on their morphology, growth condition and specific gene expression.
Mycoplasma contamination	HUVEC were tested with a MycoAlert Myplasma Detection Kit (LT07-318) and no mycoplasma contamination was found.
Commonly misidentified lines (See ICLAC register)	No commonly misidentified cell lines were used.

Animals and other organisms

Policy information about studies involving animals; ARRIVE guidelines recommended for reporting animal research

Laboratory animals	All mouse lines used in this study have been described (all noted in the text):<br>1. VE-cadherin-Cre-ERT2 mice<br>2. R26-tdTomato reporter mice<br>3. MST1 floxed mice<br>4. MST2 null mice<br>5. Foxo1 floxed mice
Wild animals	N/A
Field-collected samples	N/A
Ethics oversight	We complied with all ethical regulations for animal testing and research and performed all animal experiments under the approval from the Institute Animal Care and Use Committee (No. KA2017-31) of KAIST.

Note that full information on the approval of the study protocol must also be provided in the manuscript.

Flow Cytometry

Plots

Confirm that:

☒ The axis labels state the marker and fluorochrome used (e.g. CD4-FITC).

☒ The axis scales are clearly visible. Include numbers along axes only for bottom left plot of group (a 'group' is an analysis of identical markers).

☒ All plots are contour plots with outliers or pseudocolor plots.

☒ A numerical value for number of cells or percentage (with statistics) is provided.

Methodology

Sample preparation	Lung was harvested at P6, cut into small pieces, and digested in buffer containing collagenase type 2 (Worthington), Dispase (Gibco, 17105041) and DNase I (Roche) at 37°C for 30 min. Tissues were gently agitated, strained with a 100 µm nylon mesh to remove cell clumps, incubated in ACK lysis buffer for 2 min to remove erythrocytes and strained with a 40 µm nylon mesh again.
Instrument	Cell sorting was performed with FACSAriaII (BD Biosciences).
Software	FACS DIVA and FlowJo software were used for data analyses.
Cell population abundance	2,000,000 cells in each CD31+ lung endothelial cells and CD31- lung non-endothelial cells were captures in all experiments.
Gating strategy	The gating strategy is described in the figure.<br>1. FCS/SSC gating to discard debris, then DAPI/SSC to select alive cells.<br>2. CD45-FITC/TER119-FITC to discard hematopoietic cells.<br>3. CD31-APC/SSC to visulaize CD31+ EC and CD31- Non-EC.

☒ Tick this box to confirm that a figure exemplifying the gating strategy is provided in the Supplementary Information.
